# Patient-Specific Finite Element Analysis of Tibialis Anterior Tendon Insertion Variability and Its Impact on First Ray Biomechanics

**DOI:** 10.3390/bioengineering13040389

**Published:** 2026-03-27

**Authors:** Recep Taşkin, İrfan Kaymaz, Osman Yazici, Fatih Ugur

**Affiliations:** 1 Department of Orthopedics and Traumatology, Faculty of Medicine, Kastamonu University, 37150 Kastamonu, Turkey; fugur@kastamonu.edu.tr; 2 Department of Mechanical Engineering, Faculty of Engineering and Architecture, Erzurum Technical University, 25050 Erzurum, Turkey; irfan.kaymaz@erzurum.edu.tr (İ.K.); osman.yazici@erzurum.edu.tr (O.Y.)

**Keywords:** hallux valgus, tibialis anterior, tendon insertion, finite element analysis, first ray biomechanics, medial column

## Abstract

Background: Hallux valgus (HV) is a complex forefoot deformity influenced by interactions between osseous alignment, ligamentous restraint, and muscle–tendon forces. While the biomechanical role of ligament laxity and bone geometry has been extensively investigated, the contribution of tibialis anterior (TA) tendon insertion variability to medial column mechanics remains insufficiently understood. Materials and Methods: A patient-specific finite element model of the foot was developed from high-resolution computed tomography data. Five anatomically documented TA distal insertion configurations were modeled, representing different distributions of attachment to the medial cuneiform and first metatarsal base. All simulations were performed under identical boundary and loading conditions representative of the stance phase of gait. Global (full-foot) and local (first bone and first metatarsal) mechanical responses were quantified using total deformation, equivalent von Mises stress, and strain distributions. Results: Marked differences in mechanical behavior were observed across TA insertion types. The metatarsal-dominant configuration (Type 3) demonstrated the highest global and local deformation values (global deformation: 1.0928 mm; first bone deformation: 1.0928 mm) and elevated strain distributions, whereas the medial-dominant configuration (Type 2) showed minimal deformation (global: 0.0727 mm; first bone: 0.0350 mm) but the highest global equivalent von Mises stress (5.7698 MPa). The single-band insertion to the medial cuneiform (Type 5) produced the greatest localized stress in the first bone region (3.8634 MPa). Representative strain maps revealed distinct spatial redistribution patterns within the medial column associated with TA insertion geometry. Conclusions: This patient-specific finite element analysis indicated that distal TA insertion variability alone can substantially modify deformation, stress, and strain patterns within the medial column. These findings suggested that TA insertion anatomy may act as a biomechanical modulator of first-ray mechanics and should be considered in future studies investigating hallux valgus pathomechanics and personalized treatment strategies.

## 1. Introduction

Hallux valgus (HV) is one of the most prevalent forefoot deformities in adults and represents a major source of pain, functional limitation, and reduced quality of life. The deformity is characterized by lateral deviation of the hallux and medial deviation of the first metatarsal, resulting in progressive incongruence of the first metatarsophalangeal (MTP1) joint [1,2]. From a biomechanical perspective, HV progression is closely linked to altered load transmission through the first ray, medial column instability, and increased deforming moments acting across the MTP1 joint during stance and push-off phases of gait [3,4,5]. These changes ultimately shift plantar load laterally, impair propulsion efficiency, and predispose the joint to degenerative changes.

Although HV has traditionally been described in terms of osseous alignment parameters such as the hallux valgus angle (HVA) and intermetatarsal angle (IMA), accumulating evidence indicates that soft tissue structures and muscle–tendon forces play a critical role in deformity initiation and progression [6,7,8]. Finite element analysis (FEA) studies have demonstrated that reductions in ligament stiffness around the first ray markedly increase deforming moments at the MTP1 joint and disrupt physiological load sharing between the medial and lateral forefoot [3,9,10,11]. These findings emphasize that HV is not merely a static bony deformity but a dynamic disorder driven by the interaction between skeletal geometry, ligamentous restraint, and muscle-generated forces.

Patient-specific FEA has emerged as a powerful tool for investigating these complex interactions in HV [12,13,14]. By integrating subject-specific bone geometry, material properties, and physiologically relevant loading conditions, FEA enables quantitative assessment of stress distribution, joint contact mechanics, and deformation patterns that are not accessible through clinical imaging alone [15,16,17,18]. Prior studies have shown that relatively small anatomical variations—such as differences in proximal phalanx geometry—can substantially alter stress concentrations and contact mechanics at the MTP1 joint, potentially influencing deformity progression [19,20,21]. Similarly, external factors such as footwear constraints have been shown to exacerbate medial joint stresses and deforming moments, further highlighting the sensitivity of MTP1 biomechanics to both intrinsic and extrinsic determinants [22].

Despite these advances, the contribution of extrinsic muscle–tendon units—particularly the tibialis anterior (TA) tendon—to first-ray and MTP1 biomechanics remains insufficiently characterized [23,24]. The TA plays a key role in dorsiflexion and inversion of the foot and contributes to dynamic stabilization of the medial longitudinal arch and first ray during gait. Anatomically, the distal insertion of the TA tendon exhibits notable interindividual variability, attaching to the medial cuneiform, the base of the first metatarsal, or both, in different configurations [25]. Recent magnetic resonance imaging studies have suggested that certain TA insertion patterns, especially those with predominant attachment to the medial cuneiform, may be associated with increased HV severity [26,27,28,29]. These observations raise the possibility that TA insertion anatomy and force transmission characteristics may modulate deforming or protective moments acting on the MTP1 joint.

However, current HV-focused FEA literature has largely treated muscle forces as fixed background inputs or has not isolated the TA as an independent biomechanical variable. A recent systematic review of FEA studies in HV emphasized the need for more targeted investigations examining specific muscle–tendon contributions, improved methodological transparency, and clinically interpretable outcome metrics [30]. In particular, there is a lack of patient-specific computational studies that parametrically manipulate TA insertion patterns and force balance—especially relative to antagonistic muscles such as the peroneus longus—and quantify their effects on MTP1 contact mechanics and deforming moments across representative gait phases.

Addressing this gap is clinically relevant. A clearer understanding of how TA tendon anatomy and loading influence first-ray biomechanics could inform conservative strategies such as orthotic design and targeted exercise programs, as well as surgical decision-making related to soft tissue balancing and recurrence risk. From a mechanistic standpoint, isolating the TA within a controlled computational framework allows separation of its effects from confounding osseous and ligamentous variables, providing insights that are difficult to obtain in vivo.

Therefore, the aim of this study was to quantitatively investigate the biomechanical effects of tibialis anterior tendon insertion variations on first-ray and MTP1 joint mechanics using a patient-specific finite element model of the foot. By systematically varying TA insertion configuration and muscle force balance under physiologically representative loading conditions, this study seeks to elucidate how TA-related factors influence joint contact distribution, deforming moments, and first-ray kinematics. In doing so, the present work aims to contribute mechanistic evidence to the understanding of hallux valgus pathomechanics and to bridge an important gap in the existing finite element literature. It is important to emphasize that the present study does not model hallux valgus deformity itself nor evaluate angular deformity parameters such as the hallux valgus angle (HVA). Instead, the objective is to isolate and quantify how variations in tibialis anterior insertion geometry may alter medial column load transmission patterns under physiologically neutral alignment conditions. Such mechanical alterations may represent potential predisposing or protective mechanisms rather than direct deformity measurements.

## 2. Materials and Methods

### 2.1. Study Design and Imaging Data

This study was designed as a single-center, retrospective, non-interventional computational biomechanics investigation based on anonymized clinical imaging data. No new imaging, intervention, or treatment was performed for the purpose of this research. All procedures complied with the Declaration of Helsinki and relevant data protection regulations. Ethical approval was obtained from the Kastamonu University Non-Interventional Clinical Research Ethics Committee (Approval date: 18 December 2025; Decision No: 2025-73).

Anonymized foot computed tomography (CT) data from an adult individual were retrospectively selected from the institutional imaging archive. Inclusion criteria were age between 18 and 70 years, availability of high-resolution foot CT images with a slice thickness ≤ 1.0–1.25 mm, complete visualization of the first ray, first metatarsophalangeal (MTP1) joint, metatarso-sesamoid complex, and (iv) absence of clinically relevant hallux valgus deformity (hallux valgus angle < 15°, intermetatarsal angle < 9°). Exclusion criteria included prior foot or forefoot surgery, acute trauma, inflammatory arthropathy, advanced degenerative joint disease (Kellgren–Lawrence grade ≥ 3), congenital deformities, or inadequate image quality due to motion or metal artifacts.

The selected CT dataset belonged to an adult individual without radiographic evidence of hallux valgus deformity, structural abnormalities, or degenerative joint disease. This subject was chosen because the foot morphology represented a physiologically neutral alignment (HVA < 15°, IMA < 9°), allowing isolation of tibialis anterior insertion variability without confounding deformity-related geometric alterations. Since the aim of the present study was to investigate the isolated biomechanical effect of TA insertion geometry rather than patient-to-patient variability, a single anatomically normal reference model was considered methodologically appropriate for controlled comparative finite element simulations.

### 2.2. Image Processing and Three-Dimensional Reconstruction

Raw CT images were stored in Digital Imaging and Communications in Medicine (DICOM) format and imported into Mimics software v21 (Materialise, Leuven, Belgium) for segmentation and three-dimensional reconstruction. Bone structures were segmented using threshold-based techniques with Hounsfield unit values ranging from 226 to 3074 HU, while soft tissue structures were delineated using thresholds between −700 and 226 HU. Using these parameters, 28 individual bones of the foot, including all tarsals, metatarsals, phalanges, and sesamoid bones, were reconstructed with high anatomical fidelity.

**Figure 1:**
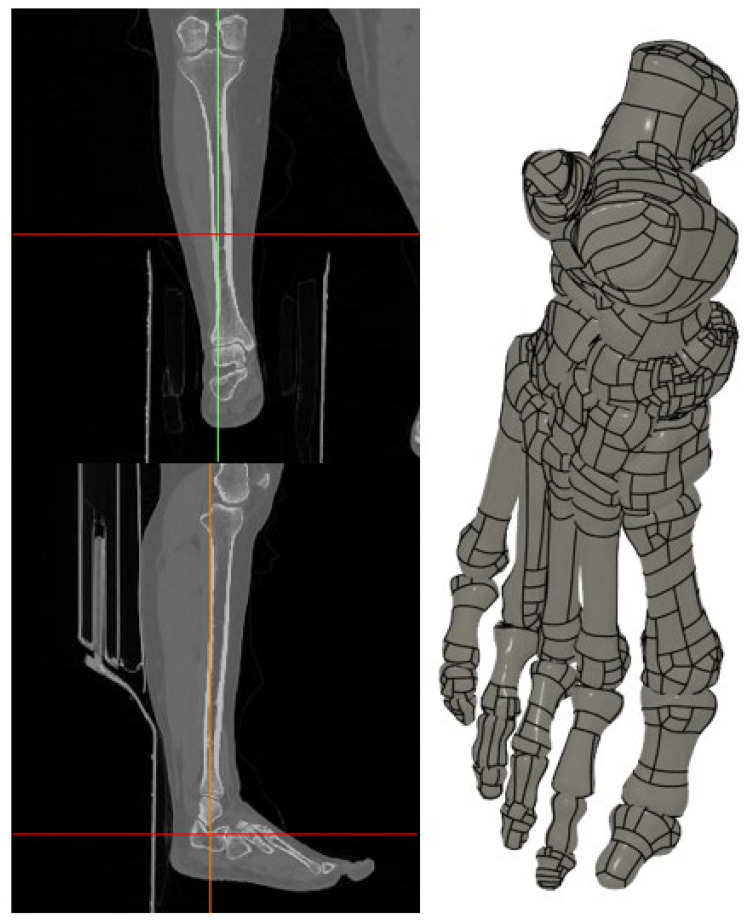
Image segmentation and three-dimensional reconstruction of the foot anatomy.

The segmented models were exported in STL format and further processed in Fusion 360 2026 (Autodesk Inc., San Rafael, CA, USA) to remove surface irregularities, optimize mesh quality, and generate watertight solid geometries suitable for finite element analysis (Figure 1).

### 2.3. Geometric Modeling of Tibialis Anterior Tendon Variations

Optimized bone and soft tissue geometries were transferred in Parasolid format to ANSYS 2025 R2 (Ansys Inc., Canonsburg, PA, USA) Design Modeler. Based on anatomical landmarks, major foot tendons—including the Achilles tendon and tibialis anterior (TA) tendon—were reconstructed using swept cross-sectional geometries.

To investigate the biomechanical influence of distal TA insertion anatomy, five distinct insertion models were constructed, reflecting clinically reported anatomical variations. The five insertion configurations were derived from previously documented anatomical classifications of tibialis anterior tendon insertion patterns reported in cadaveric and imaging-based studies [15,16]. These models were constructed to represent clinically observed morphological variability rather than hypothetical geometries.

- Type 1 (Balanced insertion): Two equal bands (diameter 7.15 mm) inserting into the medial cuneiform and the base of the first metatarsal.
- Type 2 (Medial-dominant): A larger band (7.25 mm) inserting into the medial cuneiform and a smaller band (4.26 mm) inserting into the first metatarsal base.
- Type 3 (Metatarsal-dominant): A smaller band (4.26 mm) inserting into the medial cuneiform and a larger band (7.25 mm) inserting into the first metatarsal base.
- Type 4 (Triple-band insertion): One small band (4.26 mm) inserting into the medial cuneiform and two bands (5.5 mm and 7.25 mm) inserting into the first metatarsal base.
- Type 5 (Single insertion): A single band (7.25 mm) inserting exclusively into the medial cuneiform, serving as a control configuration.

**Figure 2:**
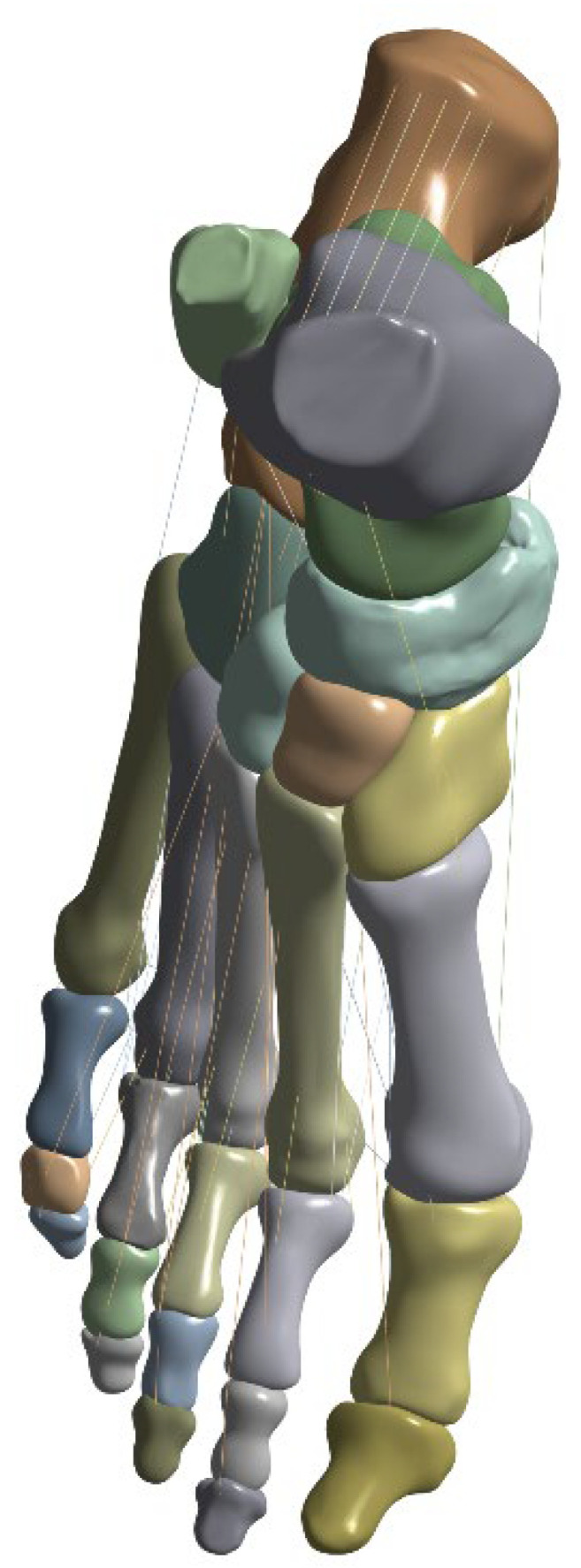
Geometric models of tibialis anterior tendon insertion variations.

Each tendon bundle was generated by sweeping a circular cross-section along an anatomically defined centerline between its origin and insertion points to preserve physiologically realistic tendon paths and cross-sectional areas, and the resulting configurations were designed to represent a spectrum of clinically observed insertion patterns and enable systematic evaluation of load transmission differences (Figure 2).

### 2.4. Material Properties and Contact Definitions

Finite element analyses were conducted using the Static Structural module of ANSYS Workbench (version 2023 R2; Ansys Inc., Canonsburg, PA, USA). All materials were assumed to be homogeneous, isotropic, and linearly elastic. Based on previously published biomechanical studies, bone structures were assigned a Young’s modulus of 10 GPa and a Poisson’s ratio of 0.30 [31]. Tendon structures were modeled with a Young’s modulus of 0.45 GPa and a Poisson’s ratio of 0.30, reflecting their compliant yet load-bearing nature [12]. Contact interactions were defined to replicate physiological behavior. Tendon-to-bone insertion sites were modeled using bonded contact conditions to prevent separation. Since articular cartilage cannot be reliably segmented from CT data due to its limited contrast, the articular interfaces between bones were modeled using a no-separation contact formulation, which allows tangential sliding while maintaining joint congruency [3]. Tendons were modeled as tension-only link (truss) elements, ensuring force transmission exclusively under tensile loading conditions [32].

### 2.5. Mesh Generation

**Figure 3:**
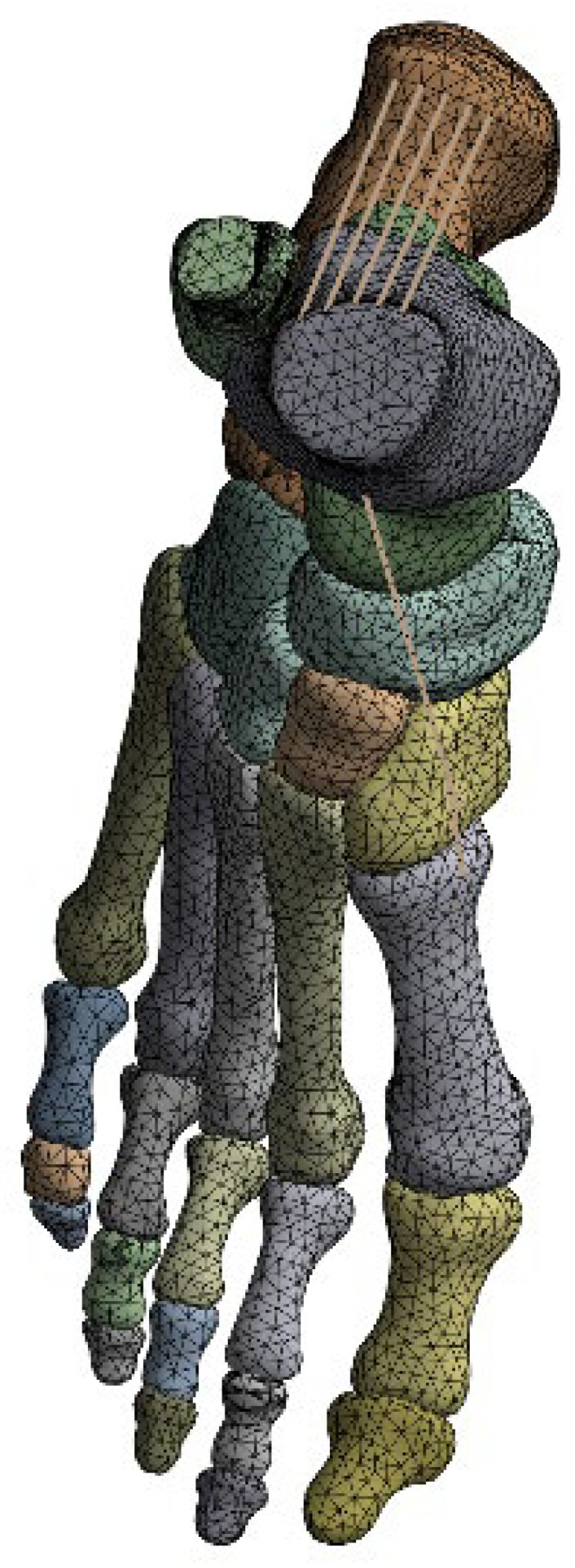
Finite element mesh structure of the foot model.

All models were discretized using predominantly tetrahedral finite elements. A global element size of 3 mm was selected as a compromise between computational efficiency and numerical accuracy. The resulting mesh consisted of approximately 86,600 elements and 137,000 nodes, with localized refinement applied in regions of high stress gradients, particularly around tendon insertion sites and the MTP1 joint (Figure 3). A formal mesh convergence study was not performed because the primary objective of the present analysis was comparative evaluation among tibialis anterior insertion configurations rather than absolute stress quantification. However, identical mesh density and element distribution were applied across all models to ensure that relative differences in deformation and stress patterns remained numerically consistent. Mesh quality was evaluated using the skewness metric, and the final mesh exhibited an average skewness of approximately 0.25, indicating good element quality and low distortion.

### 2.6. Conditions and Loading Scenarios

**Figure 4:**
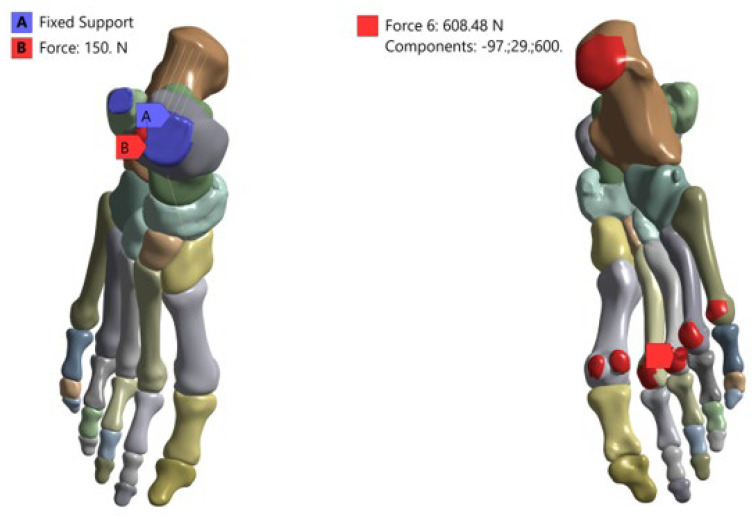
Boundary conditions and loading configuration applied to the finite element model.

To simulate the stance phase of gait, physiologically representative boundary and loading conditions were applied. The proximal ends of the tibia and fibula were fully constrained in all degrees of freedom to ensure model stability. Ground reaction forces were applied to the plantar surface of the foot using a Cartesian coordinate system, with force components defined as 97 N in the mediolateral direction, 29 N in the anteroposterior direction, and 600 N in the vertical direction, approximating body weight loading [33]. Additionally, a tensile force of 150 N was applied along the Achilles tendon axis to simulate triceps surae contribution during stance. All loading conditions were applied quasi-statically. All simulations were performed in ANSYS Static Structural 2025. The term quasi-static is used here to denote a loading assumption in which loads were applied gradually and inertial (dynamic) effects were neglected; thus, each case was solved as a static equilibrium problem rather than a transient/dynamic analysis (Figure 4).

## 3. Results

**Figure 5:**
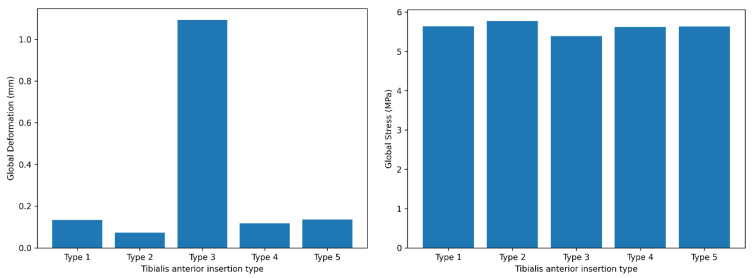
Global deformation and stress values of the full foot model across tibialis anterior insertion types.

Global (full-body) deformation and stress values across tibialis anterior insertion types are summarized in Table 1. The metatarsal-dominant configuration (Type 3) demonstrated the highest global deformation (1.0928 mm), whereas the medial-dominant configuration (Type 2) showed the lowest deformation (0.072653 mm). In contrast, the global Huber–Mises–Hencky (HMH) equivalent (von Mises) stress values exhibited a narrower range across configurations, with the highest stress observed in Type 2 (5.7698 MPa) and the lowest in Type 3 (5.3911 MPa) (Table 1, Figure 5).

**Figure 6:**
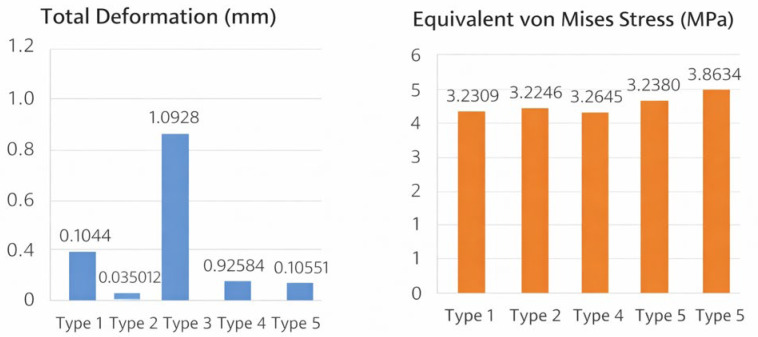
Deformation and stress distribution in the first bone across tibialis anterior insertion types.

Deformation and stress values of the first bone across tibialis anterior insertion types were shown in Table 2. Type 3 exhibited substantially higher deformation compared with all other configurations (1.0928 mm), while Type 2 showed minimal deformation (0.035012 mm). First-bone stress values were highest in the single-insertion configuration (Type 5; 3.8634 MPa) (Table 2, Figure 6).

**Figure 7:**
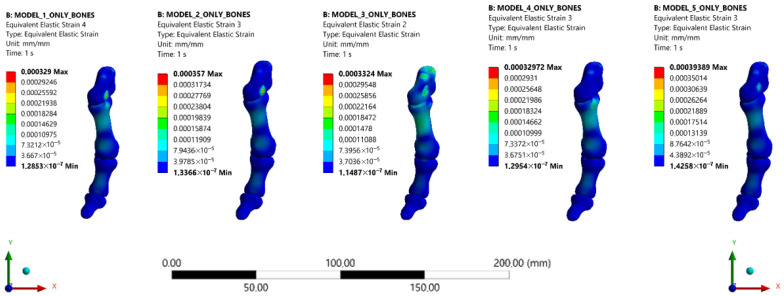
Representative equivalent strain (mm/mm) distributions in the first metatarsal and medial column across the five tibialis anterior insertion configurations (Types 1–5). Visualization is restricted to the first metatarsal and medial column to focus on biomechanically relevant regions and eliminate non-essential distal structures.

Representative equivalent strain (mm/mm) contour maps demonstrating regional strain redistribution within the medial column and first metatarsal across the five TA distal insertion configurations (Types 1–5). The metatarsal-dominant configuration (Type 3) exhibited more concentrated and higher-magnitude strain regions in the proximal first metatarsal and medial column compared with other insertion types, whereas the medial-dominant configuration (Type 2) demonstrated relatively lower and more diffuse strain patterns (Figure 7).

**Figure 8:**
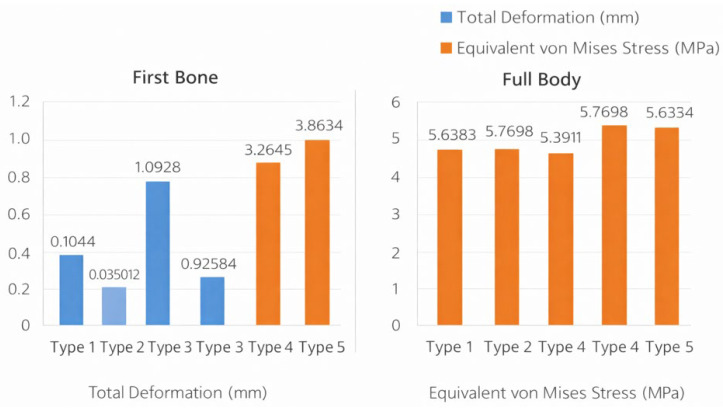
Comparative summary of deformation and stress values across tibialis anterior insertion types.

Figure 8 showed a comparative summary of total deformation and equivalent von Mises stress values across tibialis anterior insertion types for both the first bone and the full foot model. In the first bone, total deformation values ranged from 0.035012 to 1.0928 mm, while stress values ranged from 3.2246 to 3.8634 MPa across insertion types. In the full body analysis, total deformation values varied between 0.072653 and 1.0928 mm, and equivalent von Mises stress values ranged from 5.3911 to 5.7698 MPa. These values are displayed side by side to enable direct comparison of deformation and stress magnitudes across tibialis anterior insertion configurations (Figure 8).

Detailed finite element deformation distributions for the full foot model, first bone, and first metatarsal across all tibialis anterior tendon insertion types (Types 1–5) are provided in the Supplementary Material (Supplementary Figures).

## 4. Discussion

Hallux valgus is widely recognized as a multifactorial deformity influenced by osseous alignment, ligament integrity, footwear, genetic predisposition, and muscle–tendon balance. Among these factors, the tibialis anterior plays a central biomechanical role in first-ray dorsiflexion, inversion control, and medial column stabilization during gait. Unlike static osseous parameters, TA force transmission is dynamic and directly modifiable through its distal insertion geometry. Anatomical studies have consistently documented substantial interindividual variability in TA insertion patterns [15], and recent MRI-based clinical investigations have suggested associations between specific insertion types and hallux valgus severity [16]. Despite these observations, the isolated mechanical consequences of TA insertion variability have not previously been quantified using patient-specific finite element modeling. Therefore, this study focused specifically on TA insertion anatomy to address this clearly defined gap in the existing biomechanical literature.

This patient-specific FEA demonstrated that variations in distal TA tendon insertion geometry can substantially alter both global and local mechanical responses of the medial column. Three consistent patterns emerged that the metatarsal-dominant configuration (Type 3) produced markedly higher deformation and strain at both the whole-foot and first-ray levels, the medial-dominant configuration (Type 2) showed comparatively low deformation yet the highest global stress, and the single-band insertion to the medial cuneiform (Type 5) generated the highest localized stress in the first bone. Because all simulations were performed under identical boundary and loading conditions, these differences can be attributed to TA force-line orientation and insertion geometry, highlighting the tendon’s potential role as a mechanical modulator of medial column behavior.

Wong et al. demonstrated that increasing ligament laxity around the first ray amplifies deforming moments at the MTP1 and disrupts physiological load sharing [3]. While ligament properties were not varied in the present model, the elevated deformation and strain observed in Type 3 suggest that muscle–tendon force pathways, independent of ligament stiffness, can similarly destabilize medial column mechanics. This aligns with the view that HV is a dynamic deformity driven by interactions between bone geometry, soft tissues, and muscle forces rather than by static alignment alone [6,30].

Morales-Orcajo et al. reported that subtle differences in first proximal phalanx geometry significantly modify stress fields around the MTP1 joint, and Zhang et al. showed that advanced HV alters metatarsal stress distribution during stance [12,19]. Our findings are consistent with these observations, extending them by demonstrating that TA insertion geometry—without altering bone shape—can relocate stress and strain concentrations within the first ray. The originality of the present work lies in isolating TA insertion type as the primary independent variable and quantifying its mechanical impact at both global and regional levels.

Comprehensive anatomical reviews by Zielińska et al. have documented substantial variability in TA insertion patterns, including attachments to the medial cuneiform, first metatarsal base, or both [25]. By directly modeling these configurations, our results suggest that such anatomical variants are not merely descriptive but may have functional mechanical consequences. Clinical imaging studies by Uğur et al. reported associations between specific TA insertion patterns and HV severity [26]. The increased first-bone stress observed in Type 5 provides a plausible mechanical explanation for these associations, indicating that a cuneiform-focused force transfer may intensify localized loading in the medial column. Although this does not imply causation, it supports a mechanistic hypothesis linking TA anatomy to altered load environments relevant to HV progression.

The pronounced deformation and strain in Type 3 can be interpreted as a consequence of enhanced force transmission to the first metatarsal base, effectively increasing the first ray’s lever arm and altering moment balance during stance. This mechanical interpretation is supported by prior biomechanical and finite element investigations demonstrating that changes in force application site and vector orientation can significantly alter first-ray deformation and joint loading patterns. Wong et al. showed that altered load transmission pathways increase deforming moments at the MTP1 joint [2], while Morales-Orcajo et al. reported that geometric and load vector modifications substantially influence stress redistribution in hallux valgus models [5]. Similarly, Zhang et al. demonstrated that variations in loading direction can amplify metatarsal deformation during stance [10]. Therefore, the elevated deformation observed in the metatarsal-dominant configuration (Type 3) likely reflects amplified bending and torsional effects along the medial column due to distalized force transfer. Embaby et al. emphasized that the MTP1 joint experiences complex multi-axial loading, where small shifts in force orientation can substantially change contact mechanics [23]. Conversely, Type 2’s low deformation coupled with higher global stress is consistent with a stiffer load-bearing pattern, in which reduced displacement leads to stress concentration rather than energy dissipation. The localized stress peak in Type 5 suggests that restricting TA insertion to the medial cuneiform may concentrate loads proximally within the medial column, potentially influencing long-term tissue adaptation.

From a clinical perspective, these findings imply that TA insertion anatomy may influence how the medial column responds to physiological loading. This has potential relevance for conservative management (orthotic design, exercise strategies) and surgical planning, where recurrence risk and soft-tissue balance are critical considerations. Previous FEA studies have shown that footwear and external constraints can modify HV-related loading patterns [21,22]; our results suggest that patient-specific TA anatomy could further modulate these effects, supporting more individualized biomechanical approaches.

Unlike most previously published finite element studies on hallux valgus, which primarily focused on osseous geometry, ligament stiffness, or external factors such as footwear, the present study specifically isolates TA distal insertion anatomy as an independent biomechanical variable. Prior investigations by Wong et al. and others demonstrated the effects of ligament laxity and bone alignment on first-ray mechanics, but muscle–tendon insertion geometry was either held constant or not explicitly modeled. Similarly, studies by Morales-Orcajo et al. and Zhang et al. evaluated how variations in bone morphology influence stress distribution without addressing how differences in TA force transmission pathways may modulate medial column loading [12,19]. In contrast, our patient-specific model systematically compares multiple clinically documented TA insertion configurations under identical boundary and loading conditions, allowing direct attribution of observed differences in deformation, stress, and strain to TA insertion geometry alone. This approach provides a novel mechanistic perspective linking anatomical TA variants—previously described mainly in imaging and anatomical studies—to quantifiable alterations in first-ray biomechanics, thereby extending existing literature beyond static structural factors to include muscle-driven modulation of hallux valgus–related mechanics.

The clinical significance of modeling distinct TA insertion types lies in their potential influence on medial column load transmission and first-ray stability. Zielińska et al. documented substantial anatomical heterogeneity in TA insertion patterns [15], and Uğur et al. reported that certain insertion configurations were associated with increased hallux valgus severity on MRI-based evaluation [16]. Although the present study does not establish a direct clinical correlation, the observed differences in deformation and stress distribution provide a plausible biomechanical explanation for these reported associations. In particular, configurations demonstrating increased medial column stress or deformation may represent mechanical environments that predispose to progressive first-ray instability over time.

A key strength of this study is the isolation of TA insertion geometry within a patient-specific FEA framework, combined with simultaneous reporting of global metrics and local strain maps. This responds to calls for clinically interpretable FEA outputs highlighted in recent systematic reviews [30]. Because articular cartilage cannot be reliably segmented from standard CT data, cartilage layers were not modeled explicitly; instead, joint articulation was represented using a ‘no-separation’ contact formulation to prevent non-physiological gapping while allowing tangential sliding and maintaining joint congruency. Although this simplification may affect the absolute magnitude of local joint contact stresses, identical contact assumptions were applied to all models; therefore, the comparative trends among TA insertion configurations are expected to remain reliable. Although the mesh exhibited good quality (average skewness ≈ 0.25), a formal mesh-convergence study was not performed; therefore, absolute peak stress/contact values may be mesh-dependent, while comparative trends are expected to remain robust due to the identical meshing strategy applied across all models.

Limitations include the use of a single subject, quasi-static loading, and simplified material assumptions. While these are common in comparable FEA studies [3,12,31], future work should incorporate multiple subjects, dynamic gait-phase loading, and experimental validation (e.g., plantar pressure or imaging-based comparisons) to enhance generalizability [30,33]. In addition, bone was modeled as a homogeneous, isotropic material; however, real cortical and trabecular bone exhibit direction-dependent behavior, and orthotropic/anisotropic formulations may change the absolute magnitude and local distribution of predicted stresses/strains along dominant load paths. Therefore, the present findings should be interpreted primarily in terms of comparative trends among tibialis anterior insertion configurations, and future work will incorporate direction-dependent and/or CT-density–based material mapping to improve physiological fidelity.

Importantly, this study did not simulate a hallux valgus deformity model nor directly evaluate angular parameters such as HVA or IMA. Therefore, no direct correlation between tibialis anterior insertion types and clinical hallux valgus angles can be inferred. The findings should be interpreted as mechanistic insights into load redistribution patterns that may contribute to deformity progression, rather than as direct predictors of HV severity.

This study did not include patient-specific hallux valgus cases or clinical outcome data; therefore, direct correlation between tibialis anterior insertion types and clinical deformity severity cannot be inferred. Future studies combining imaging-based insertion classification with clinical angle measurements (HVA, IMA) and patient cohorts are warranted.

## 5. Conclusions

In conclusion, this patient-specific FEA indicates that distal TA insertion variability alone can shift deformation, stress, and strain patterns across the medial column. The metatarsal-dominant insertion (Type 3) was associated with disproportionate deformation and strain, whereas the single cuneiform insertion (Type 5) produced the highest localized stress in the first bone. These findings support the concept that TA insertion anatomy may act as a biomechanical modulator of first-ray mechanics and provide a mechanistic basis for future clinical and experimental investigations into its role in hallux valgus pathomechanics.

## Figures and Tables

**Table 1:** Global (full-body) deformation and stress values across tibialis anterior insertion types.

Tibialis Anterior Insertion Type	Deformation (mm)	Stress (MPa)
Type 1	0.13474	5.6383
Type 2	0.072653	5.7698
Type 3	1.0928	5.3911
Type 4	0.11744	5.6258
Type 5	0.13674	5.6334

**Table 2:** Deformation and stress values of the first bone across tibialis anterior insertion types.

Tibialis Anterior Insertion Type	Deformation (mm)	Stress (MPa)
Type 1	0.1044	3.2309
Type 2	0.035012	3.2246
Type 3	1.0928	3.2645
Type 4	0.092584	3.2380
Type 5	0.10551	3.8634

## Data Availability

The data supporting the findings of this study are available from the corresponding author upon reasonable request. The data are not publicly available due to their use in ongoing analyses and model development.

## Supplementary Materials

The following supporting information can be downloaded at: https://www.mdpi.com/article/10.3390/bioengineering13040389/s1, Supplementary Figures.

## Abbreviations

FEA	Finite Element Analysis
TA	Tibialis Anterior
MTP1	First Metatarsophalangeal Joint
HV	Hallux Valgus
CT	Computed Tomography
DICOM	Digital Imaging and Communications in Medicine
ROI	Region of Interest
MPa	Megapascal
mm	Millimeter
HU	Hounsfield Unit
IRB	Institutional Review Board
