# Supplementary material for: Patient-Specific Finite Element Analysis of Tibialis Anterior Tendon Insertion Variability and Its Impact on First Ray Biomechanics
Source: Bioengineering (Basel). 2026 Mar 27;13(4):389. doi: 10.3390/bioengineering13040389 (PMC13114066; doi:10.3390/bioengineering13040389)
Supplement: Supplementary file 1 [file bioengineering-13-00389-s001.zip › bioengineering-4163529-supplementary.pdf]

Detailed finite element deformation distributions for the full foot model, first bone, and first metatarsal across all tibialis anterior tendon insertion types (Types 1–5).

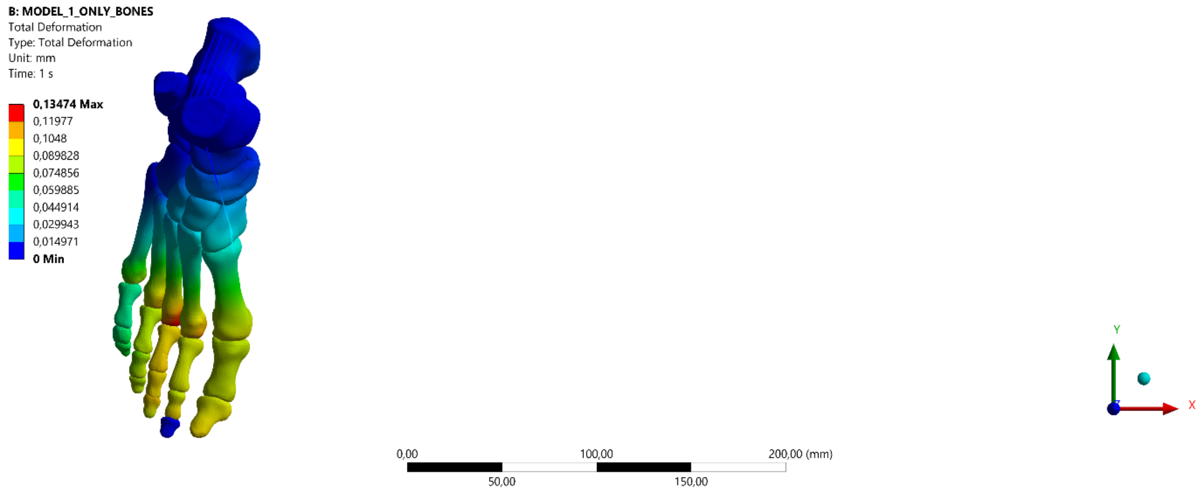

Type 1. Deformation body.

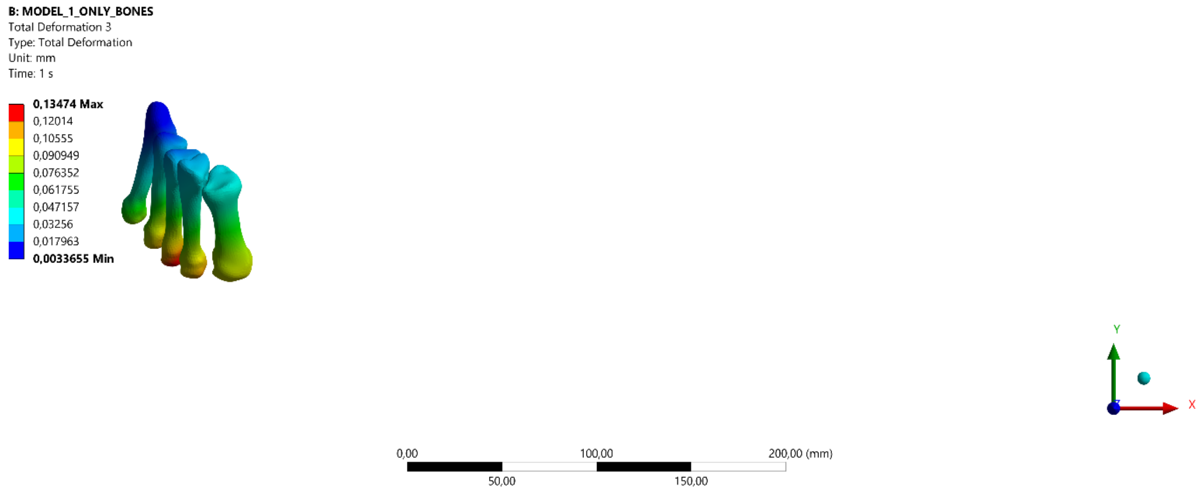

Type 1. Deformation metatars.

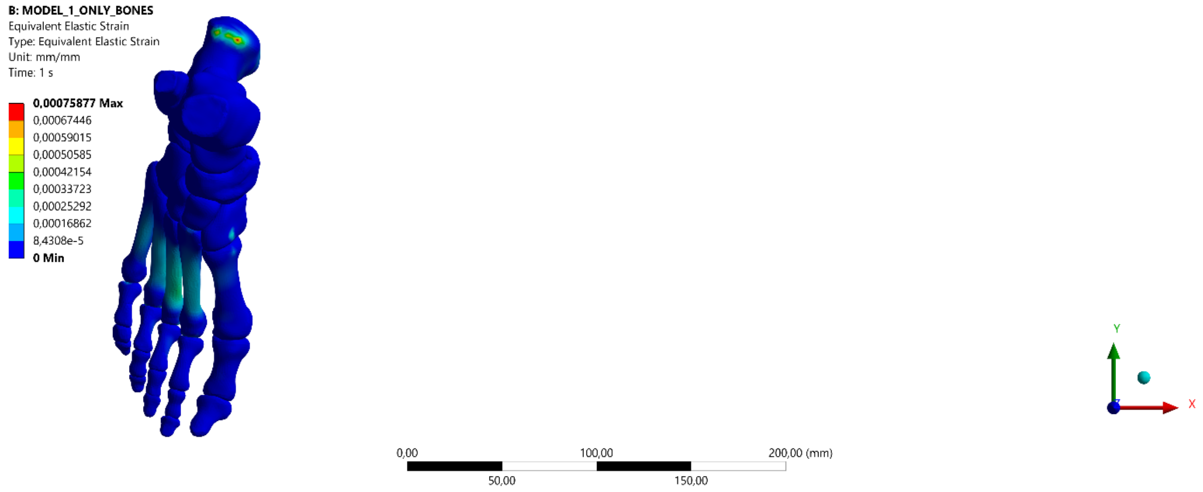

Type 1. Strain body.

Detailed finite element deformation distributions for the full foot model, first bone, and first metatarsal across all tibialis anterior tendon insertion types (Types 1–5).

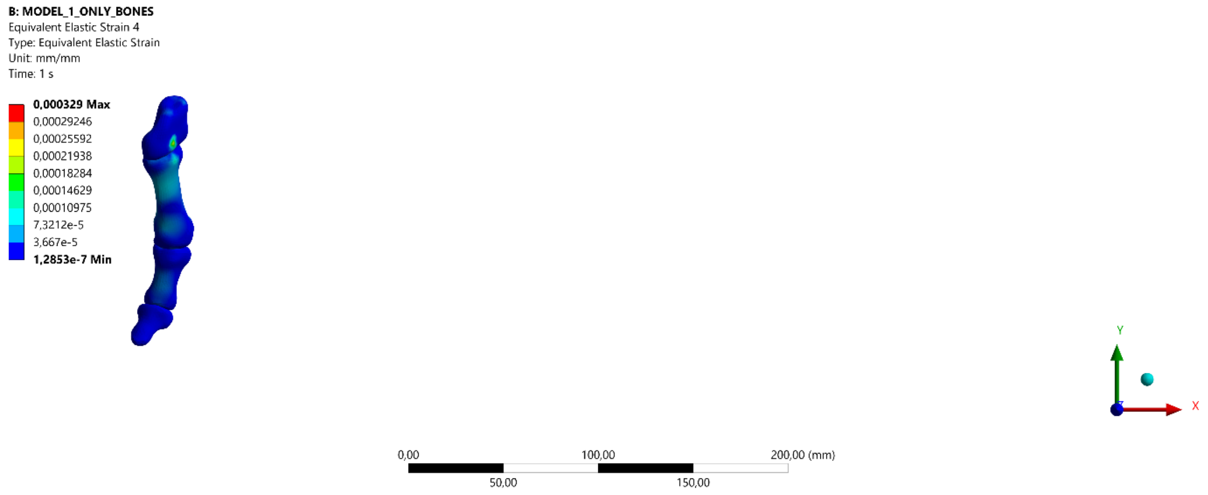

Type 1. Strain first bone.

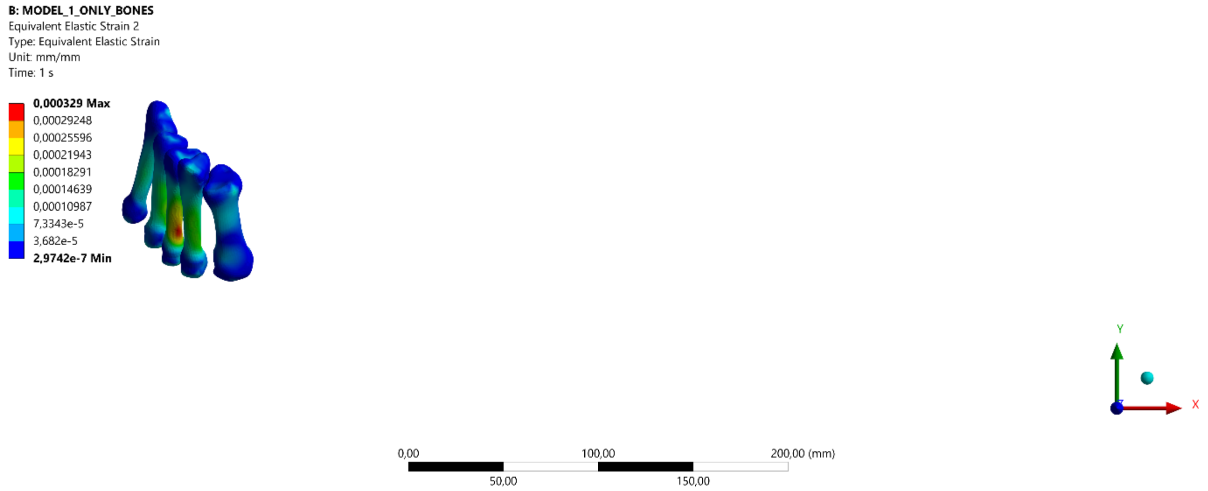

Type 1. Strain metatars.

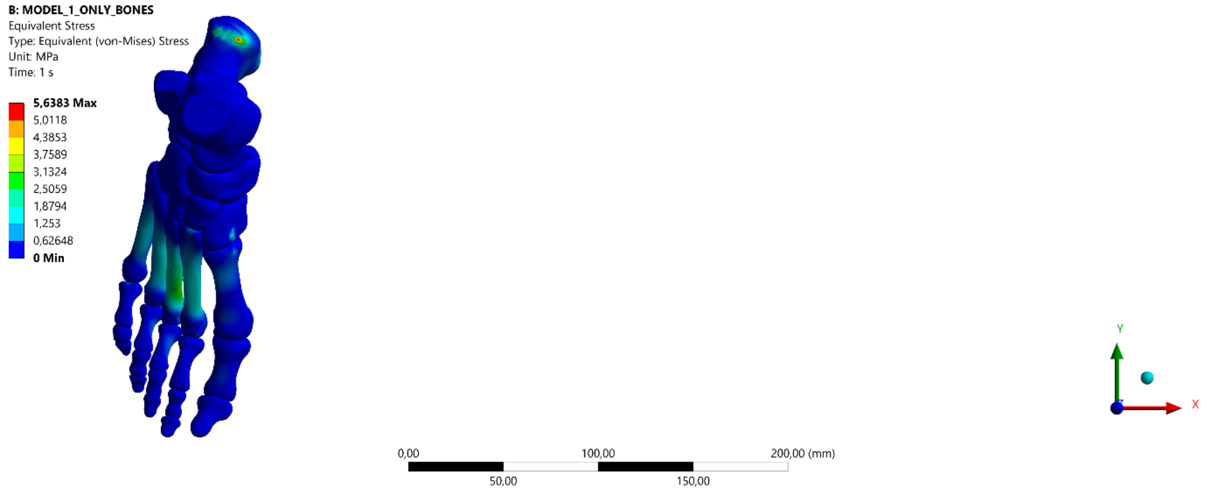

Type 1. Stress body.

Detailed finite element deformation distributions for the full foot model, first bone, and first metatarsal across all tibialis anterior tendon insertion types (Types 1–5).

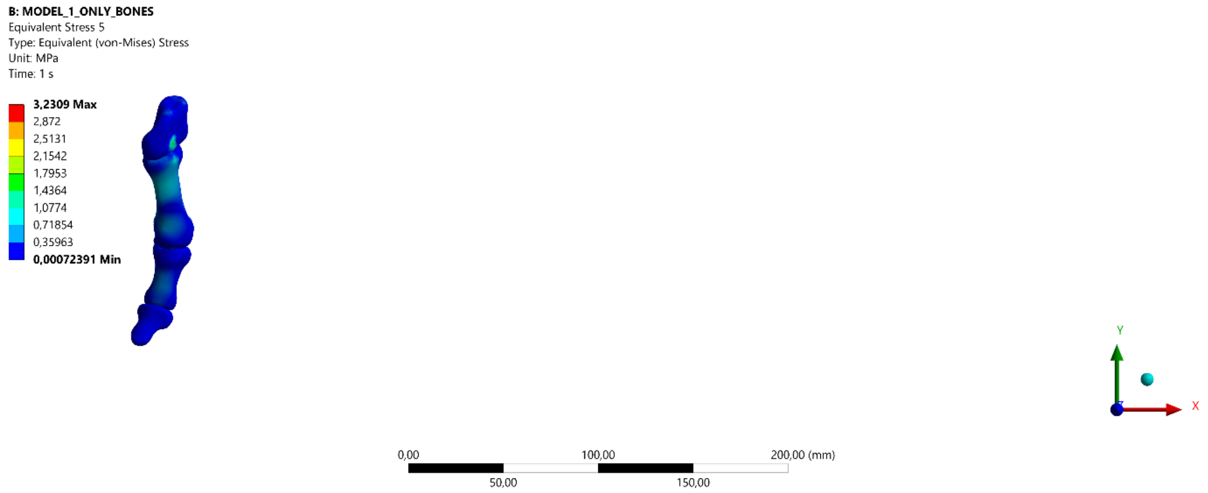

Type 1. Stress first bone.

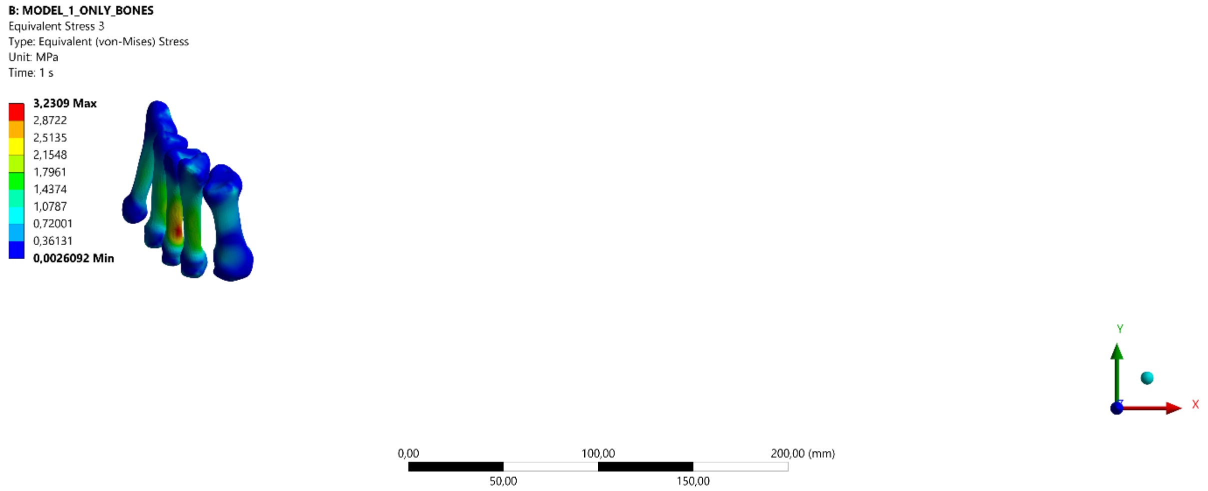

Type 1. Stress_metatars.

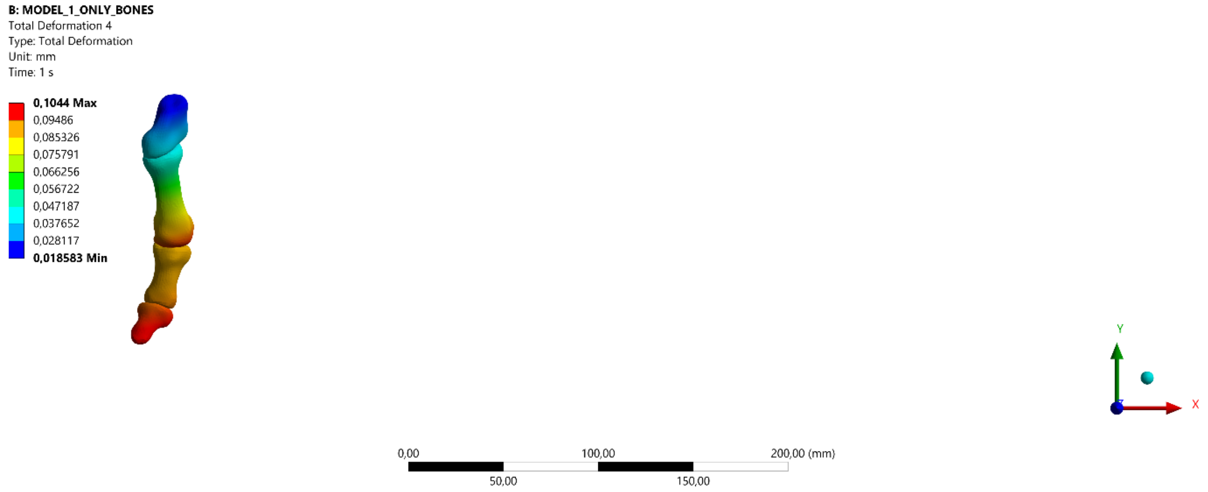

Type 1. Total deformation first bone.

Detailed finite element deformation distributions for the full foot model, first bone, and first metatarsal across all tibialis anterior tendon insertion types (Types 1–5).

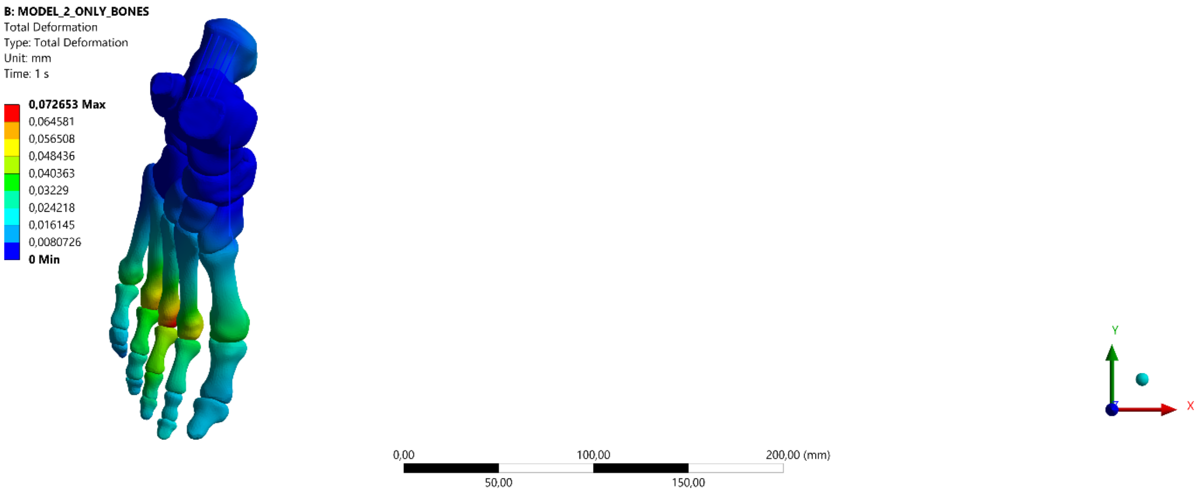

Type 2. Deformation.

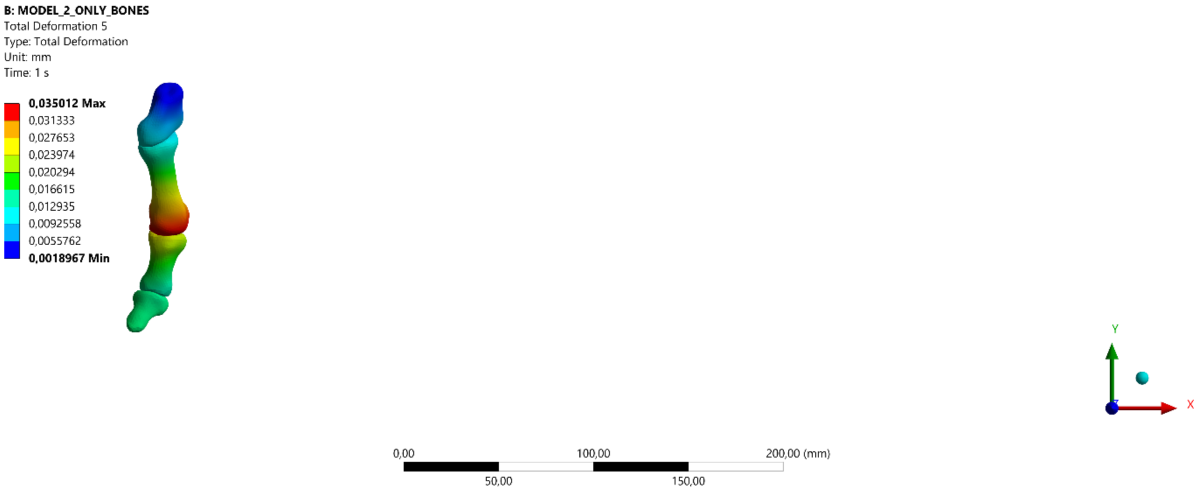

Type 2. Deformation first bone.

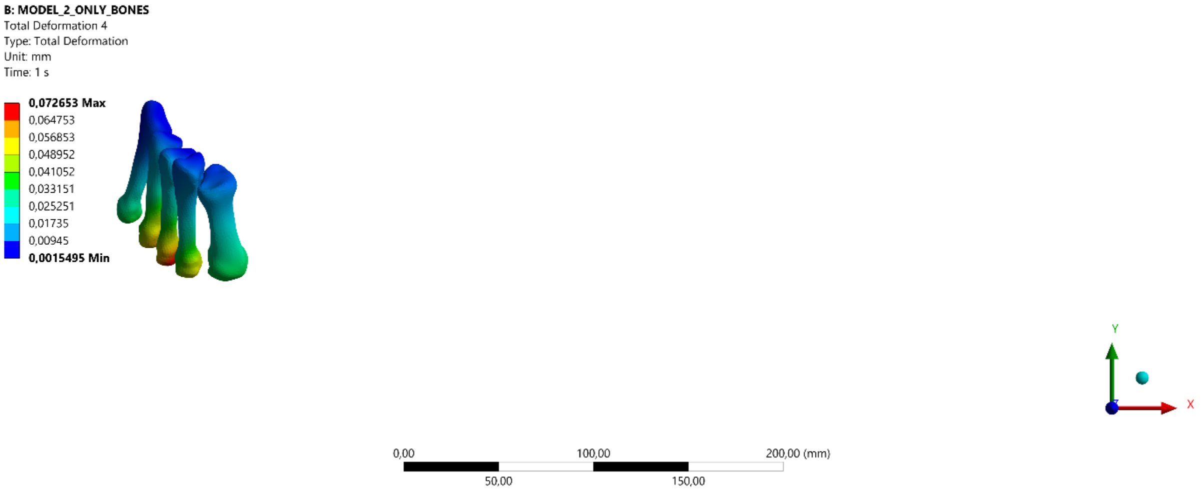

Type 2. Deformation metatars.

Detailed finite element deformation distributions for the full foot model, first bone, and first metatarsal across all tibialis anterior tendon insertion types (Types 1–5).

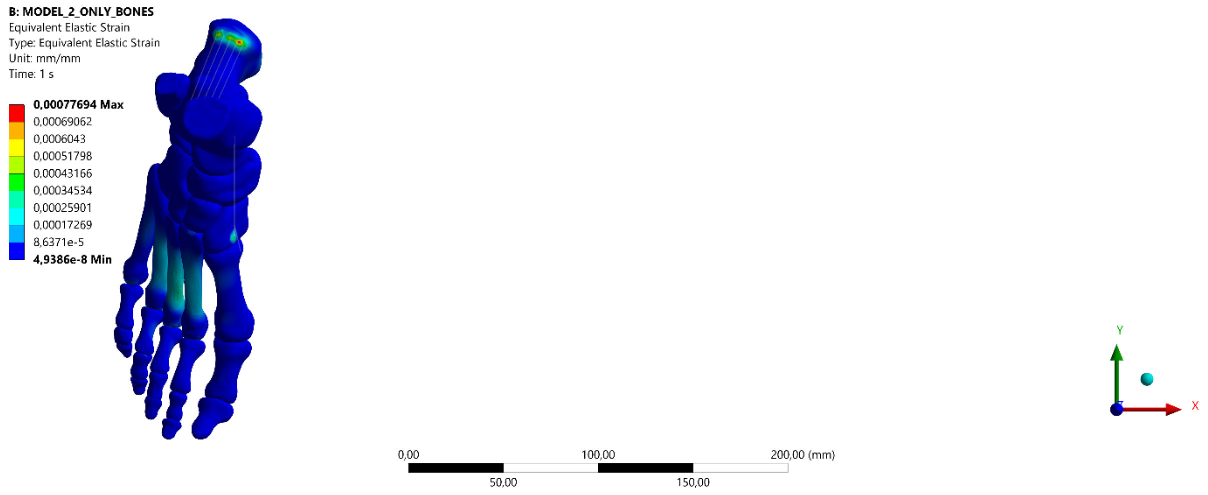

Type 2. Strain.

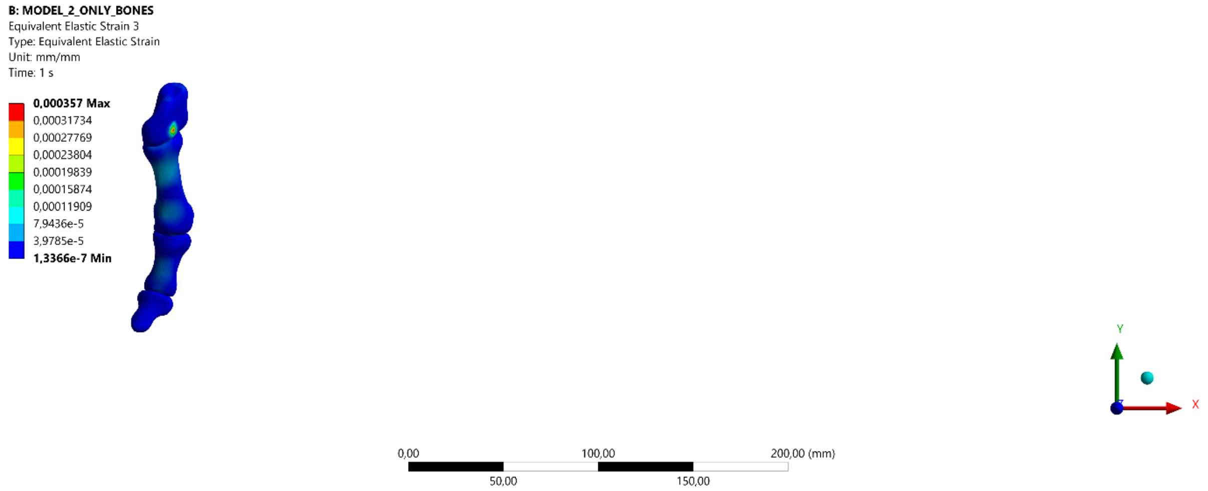

Type 2. Strain first bone.

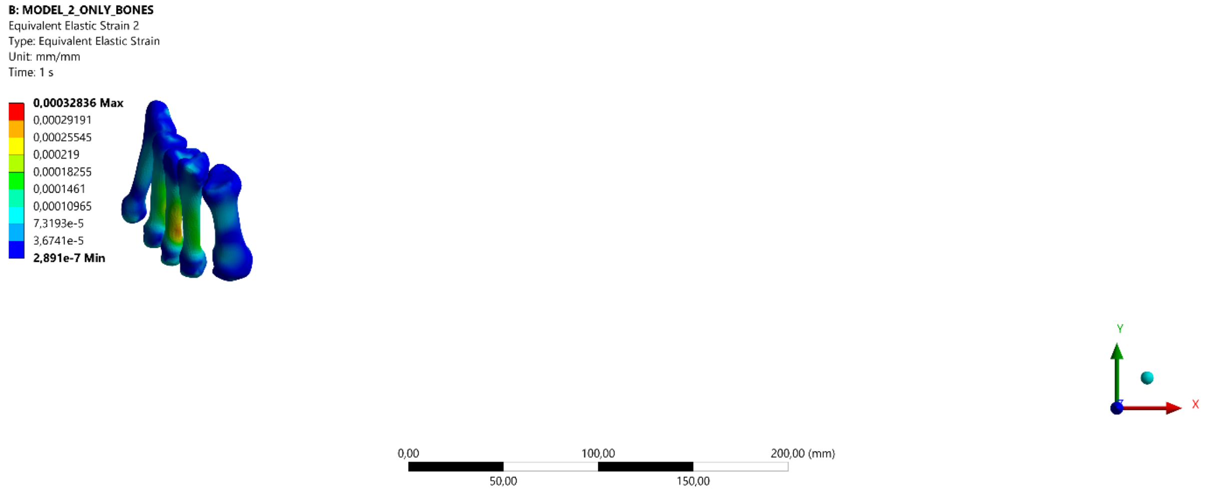

Type 2. Strain metatars.

Detailed finite element deformation distributions for the full foot model, first bone, and first metatarsal across all tibialis anterior tendon insertion types (Types 1–5).

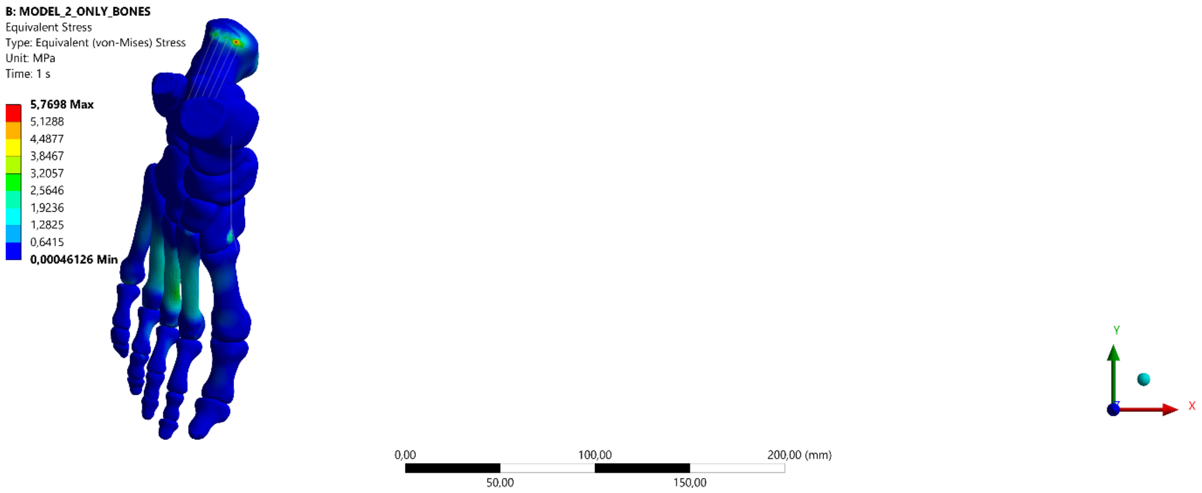

Type 2. Stress.

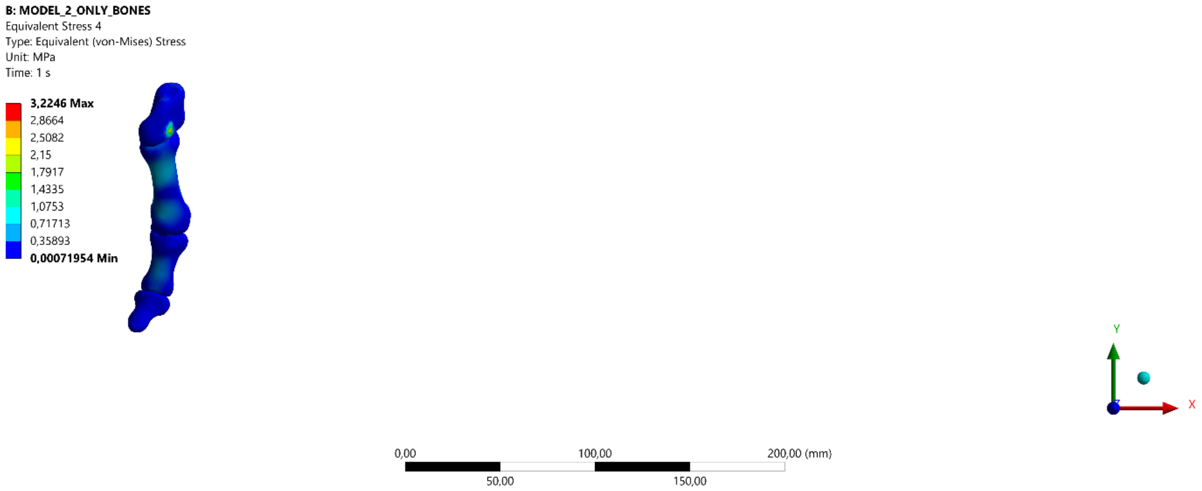

Type 2. Stress first bone.

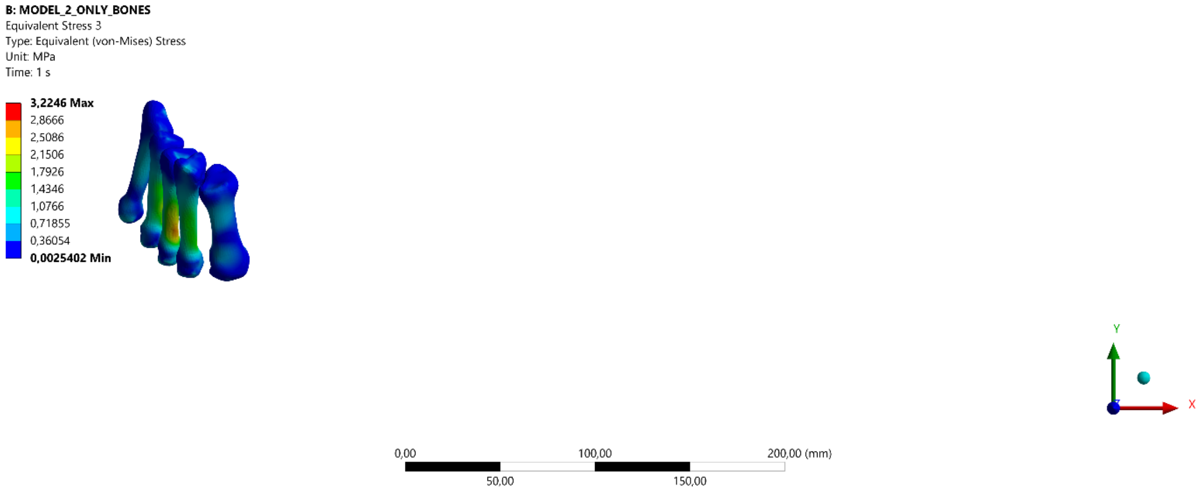

Type 2. Stress metatars.

Detailed finite element deformation distributions for the full foot model, first bone, and first metatarsal across all tibialis anterior tendon insertion types (Types 1–5).

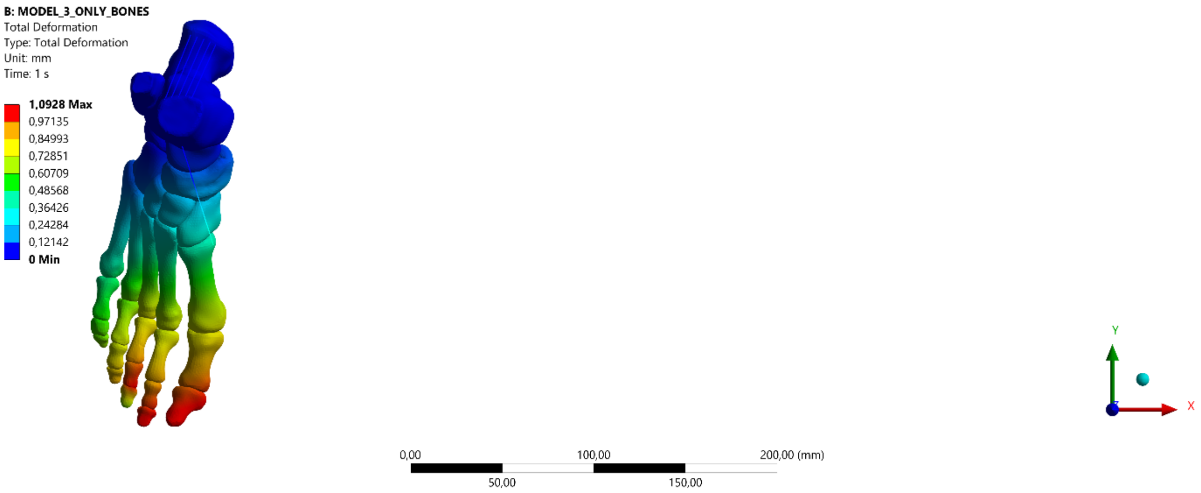

Type 3. Deformation.

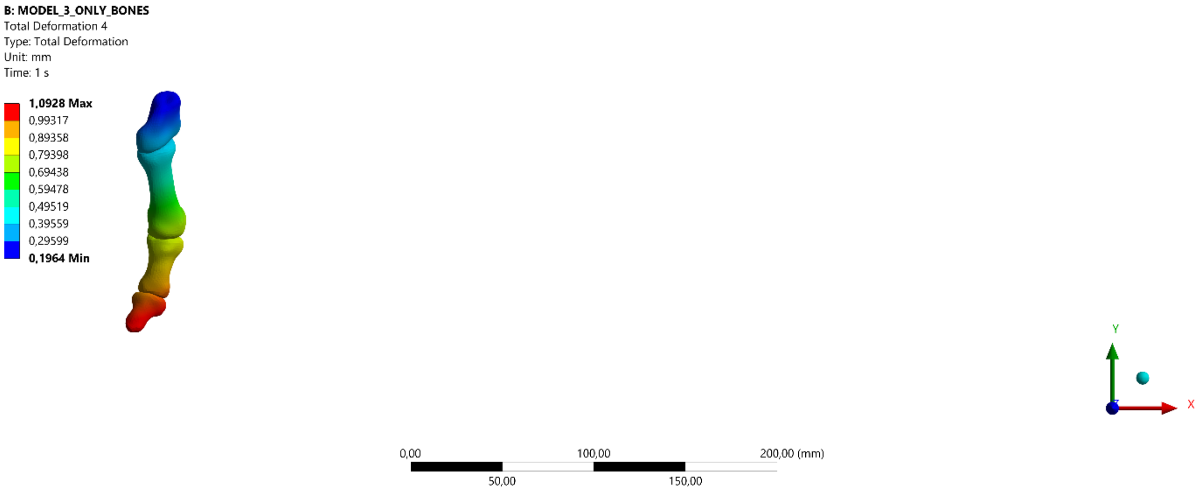

Type 3. Deformation first bone.

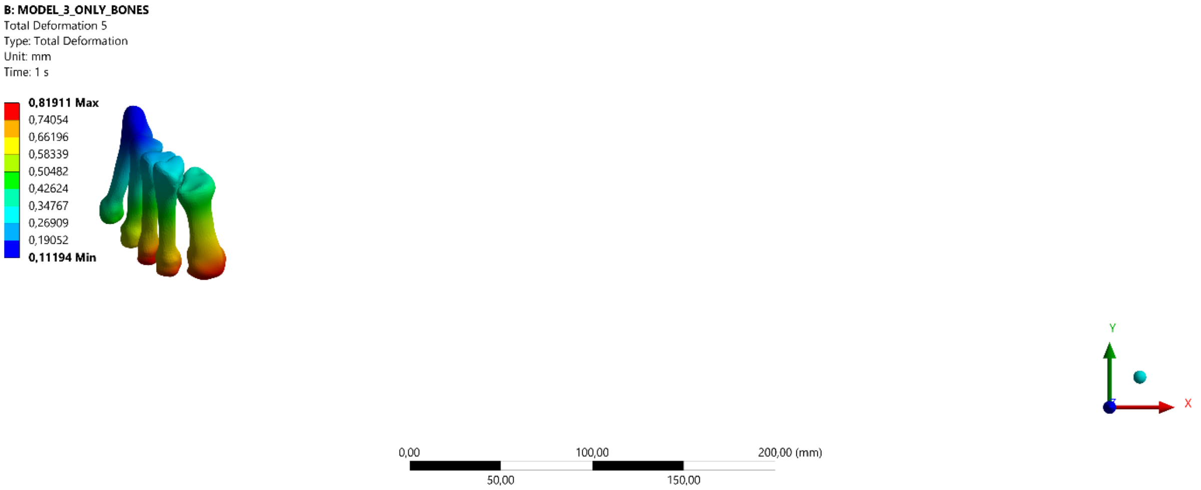

Type 3. Deformation metatars.

Detailed finite element deformation distributions for the full foot model, first bone, and first metatarsal across all tibialis anterior tendon insertion types (Types 1–5).

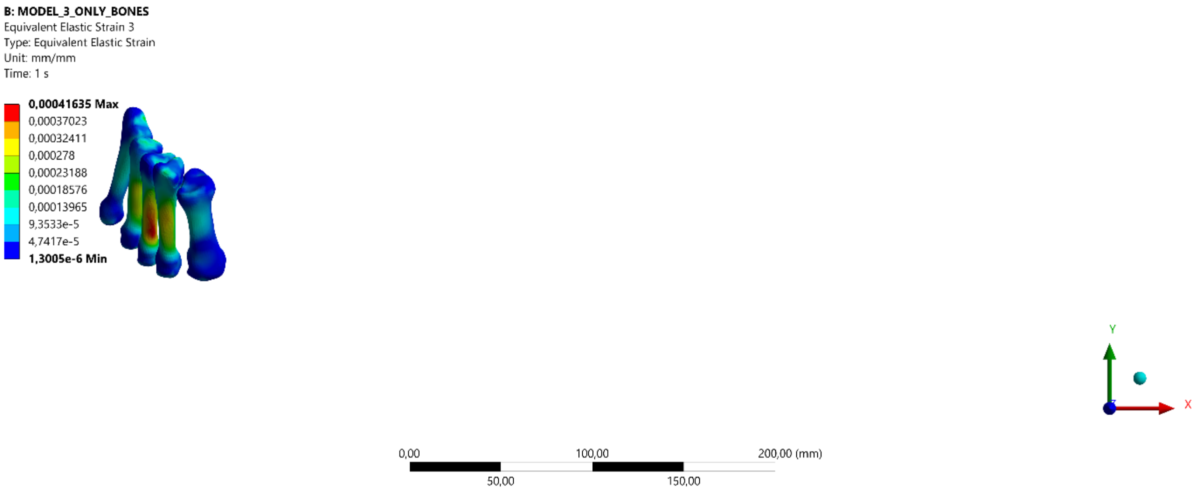

Type 3. Strain metatars.

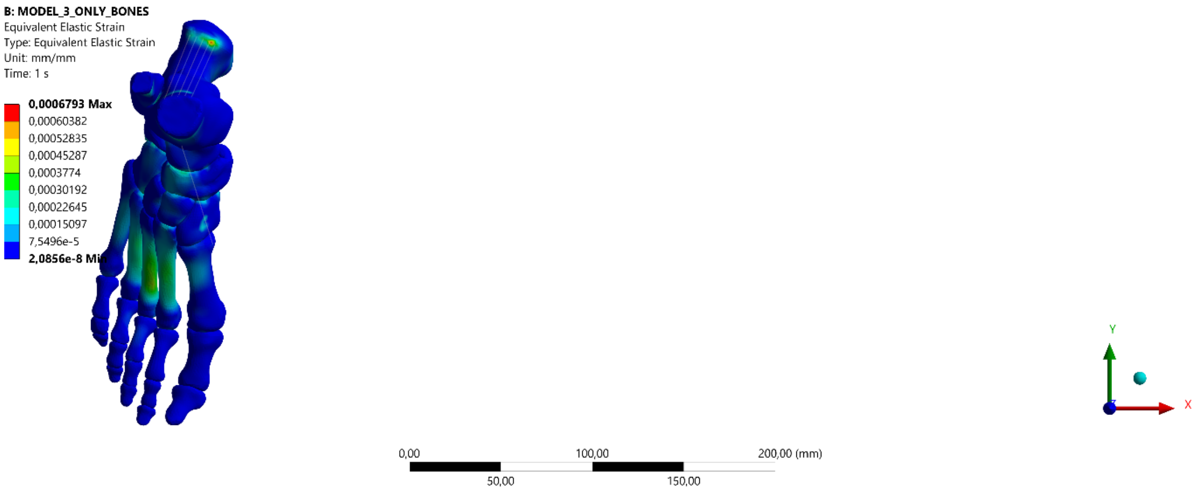

Type 3. Strain.

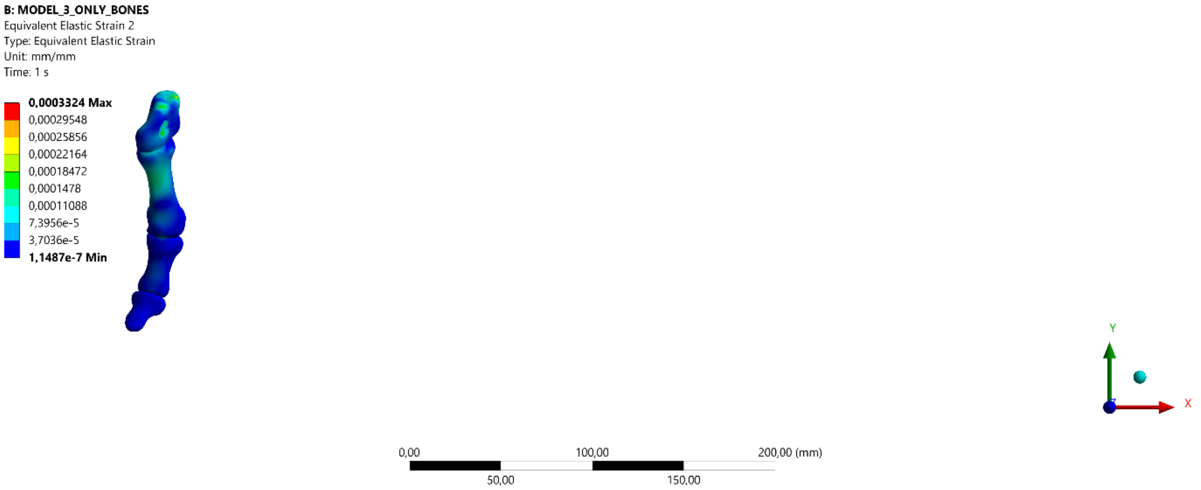

Type 3. Strain first bone.

Detailed finite element deformation distributions for the full foot model, first bone, and first metatarsal across all tibialis anterior tendon insertion types (Types 1–5).

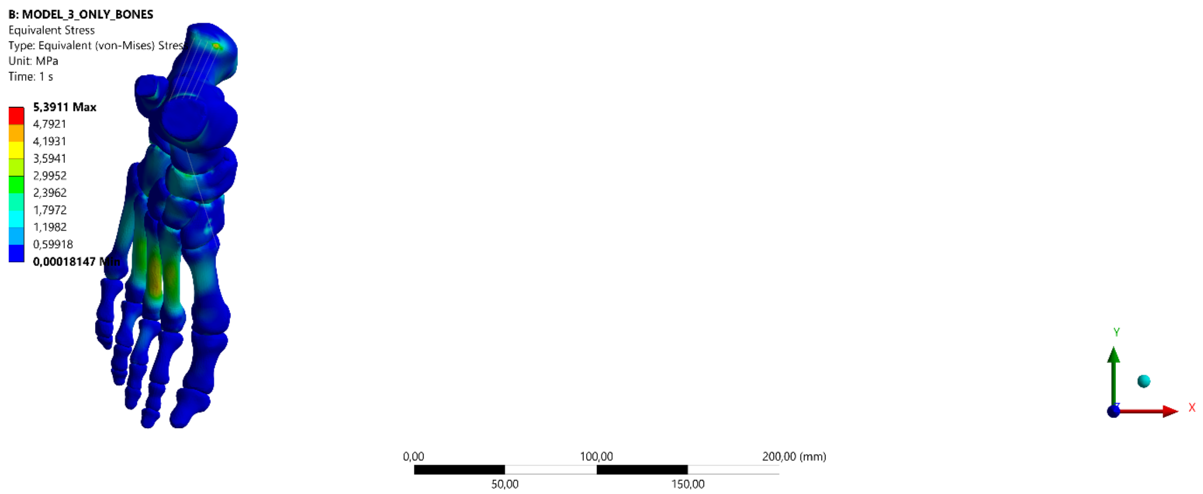

Type 3. Stress.

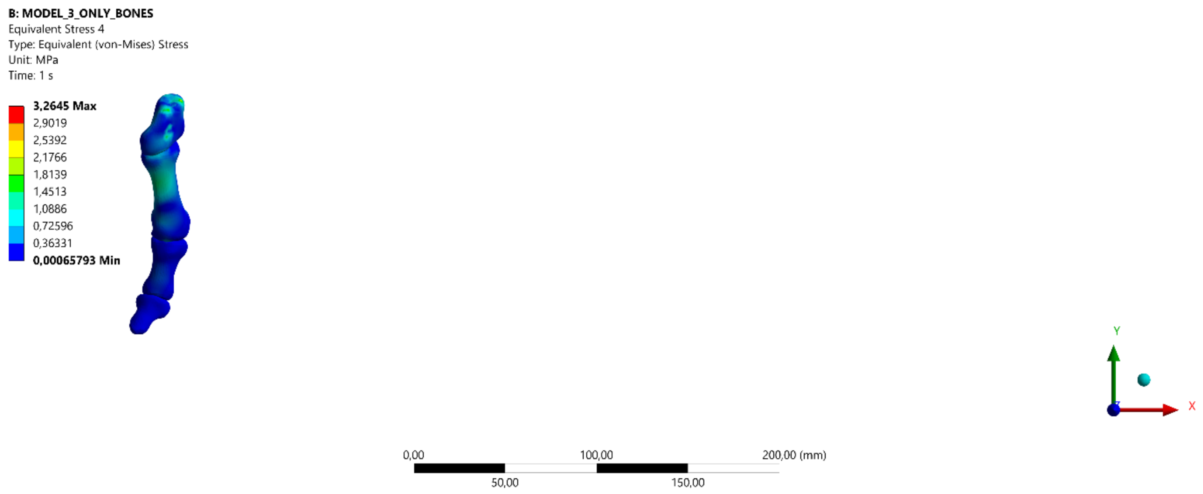

Type 3. Stress first bone.

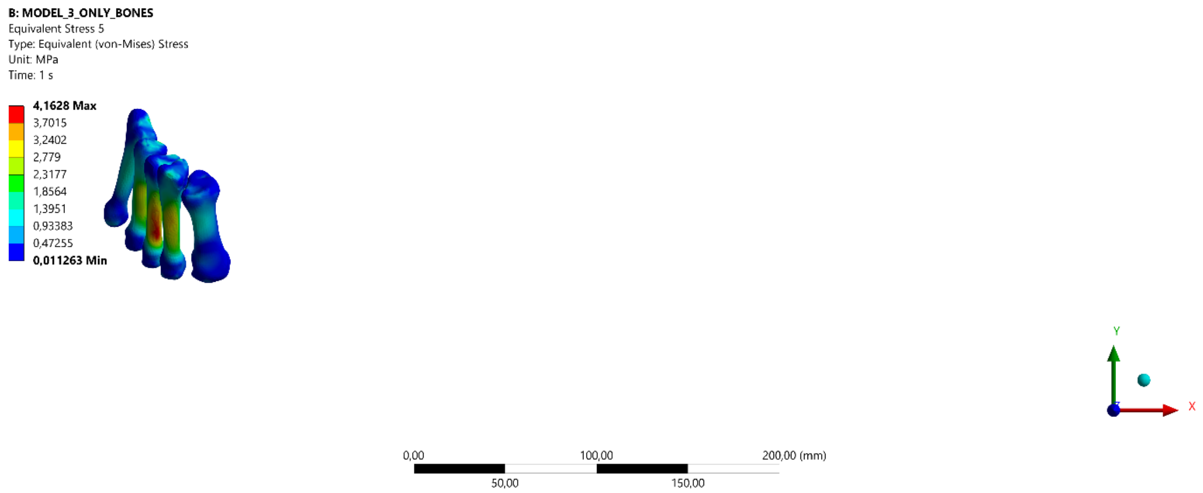

Type 3. Stress metatars.

Detailed finite element deformation distributions for the full foot model, first bone, and first metatarsal across all tibialis anterior tendon insertion types (Types 1–5).

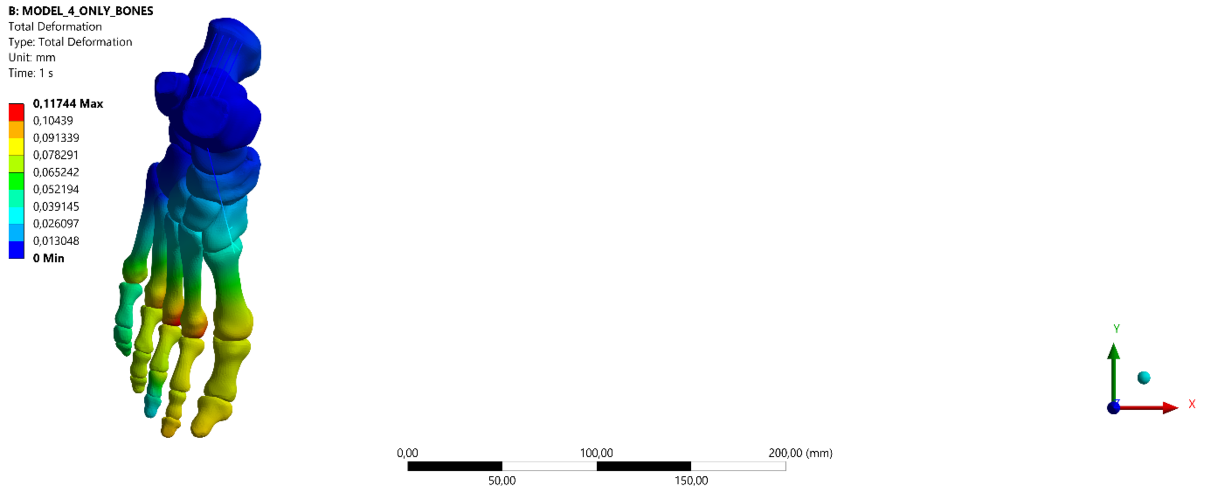

Type 4. Deformation.

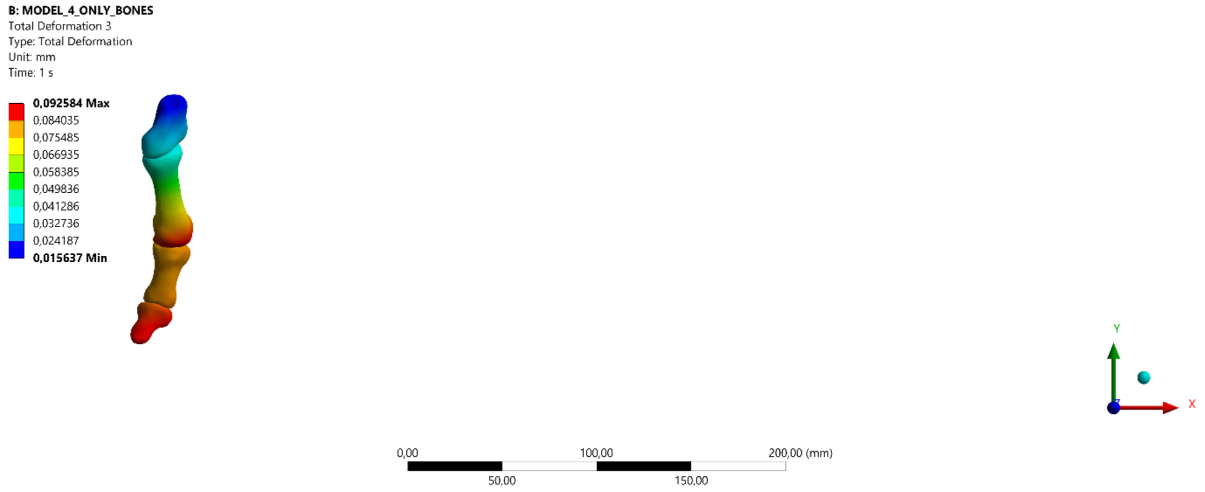

Type 4. Deformation first bone.

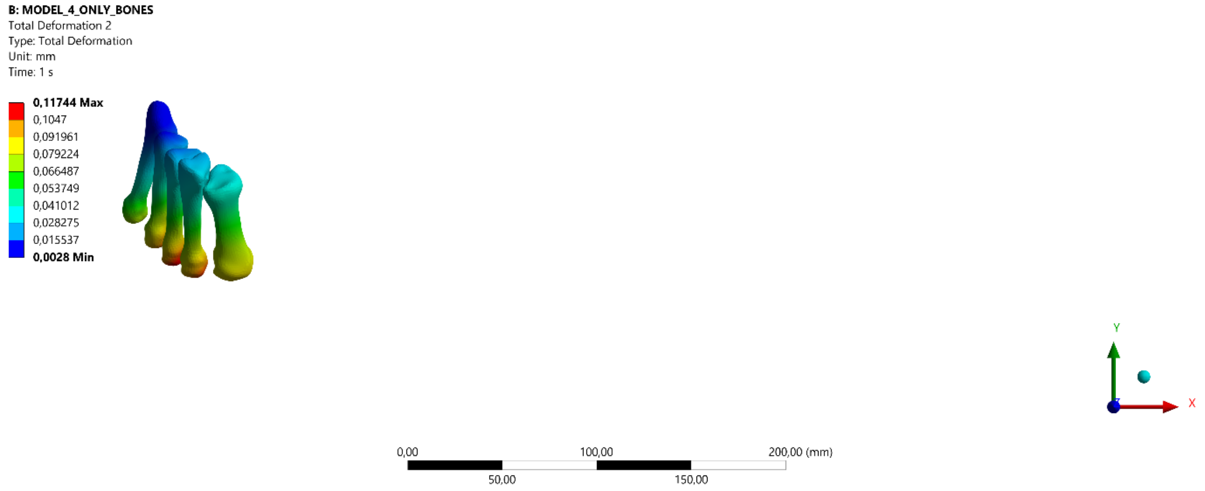

Type 4. Deformation metatars.

Detailed finite element deformation distributions for the full foot model, first bone, and first metatarsal across all tibialis anterior tendon insertion types (Types 1–5).

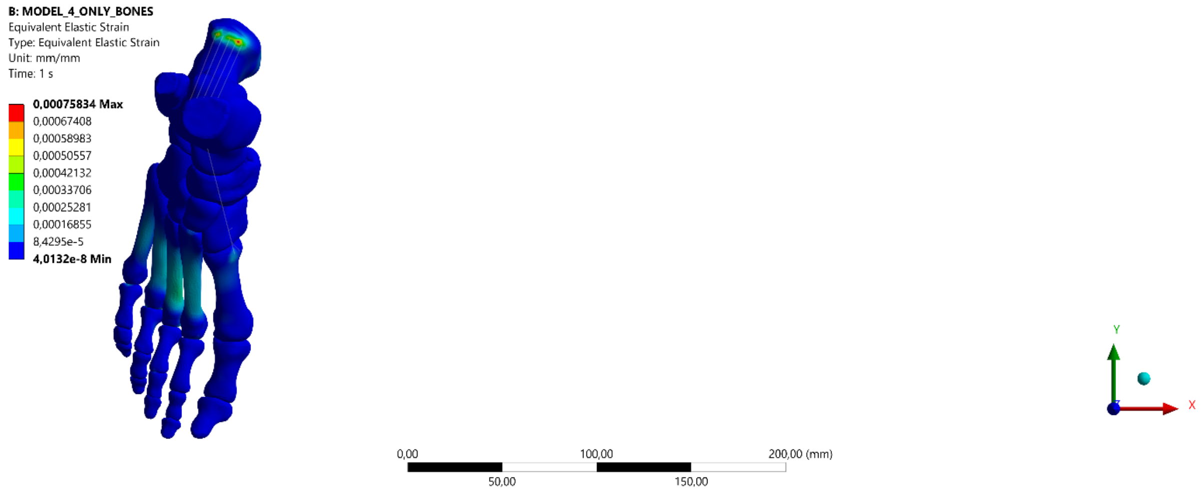

Type 4. Strain.

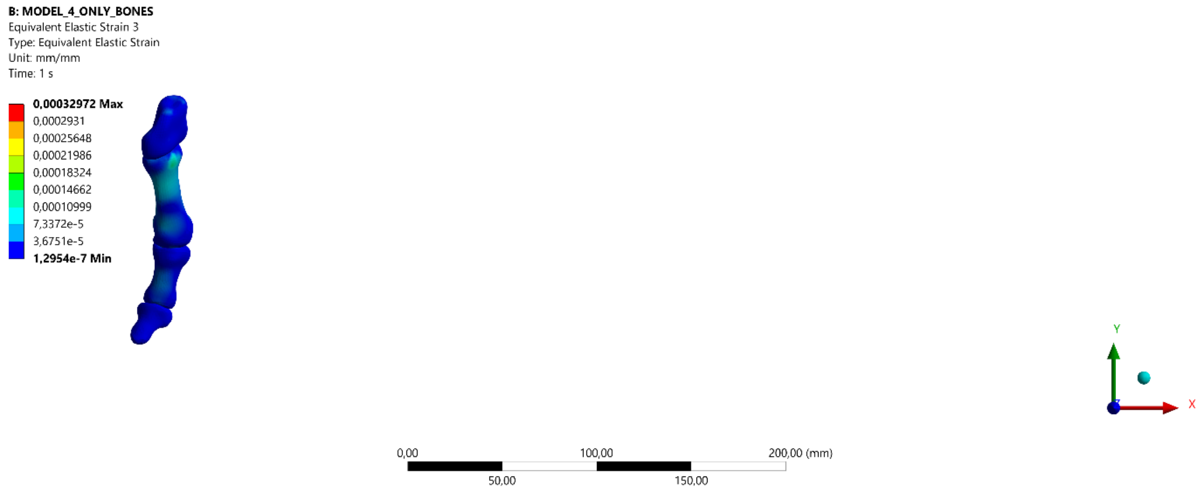

Type 4. Strain first bone.

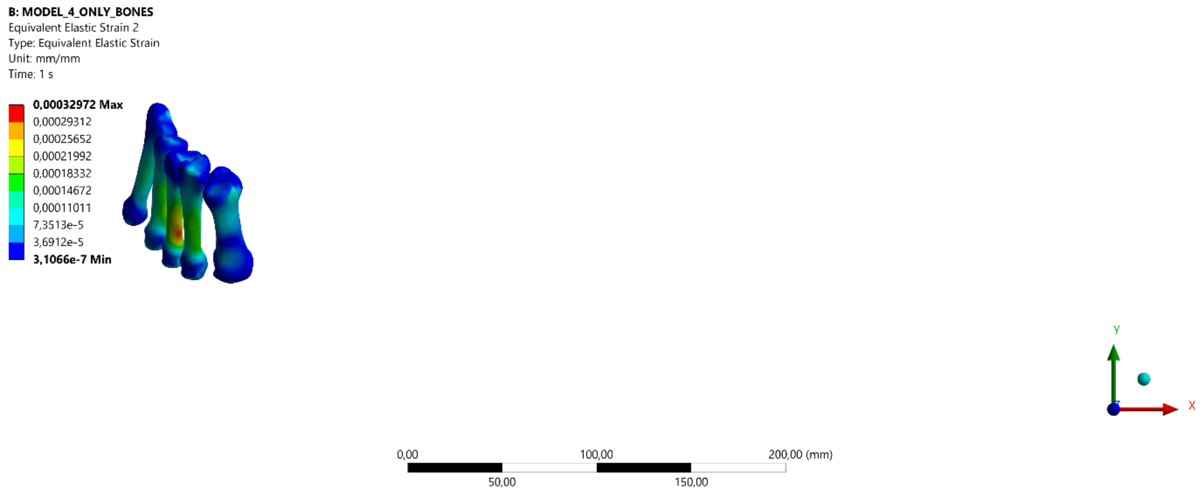

Type 4. Strain metatars.

Detailed finite element deformation distributions for the full foot model, first bone, and first metatarsal across all tibialis anterior tendon insertion types (Types 1–5).

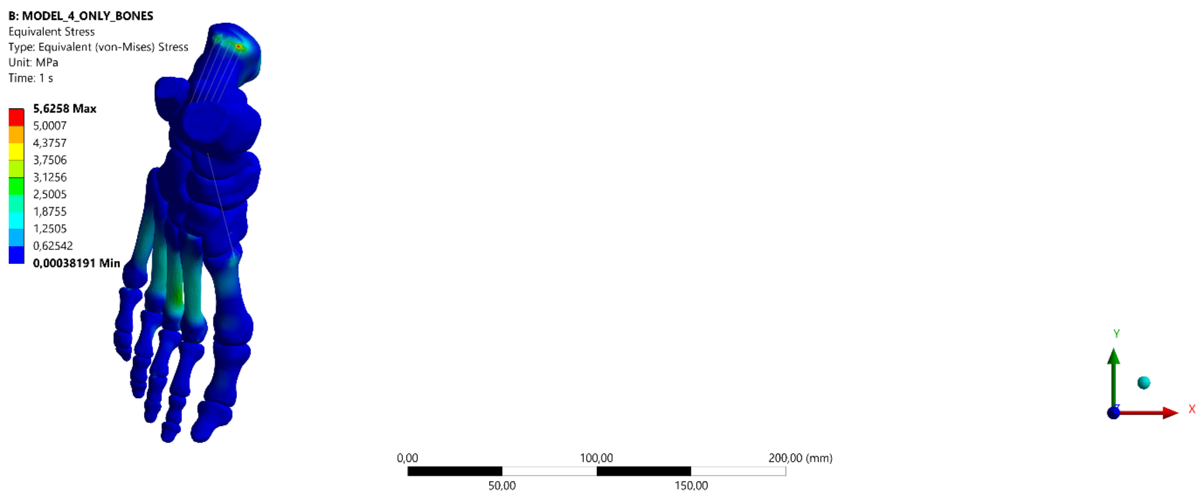

Type 4. Stress.

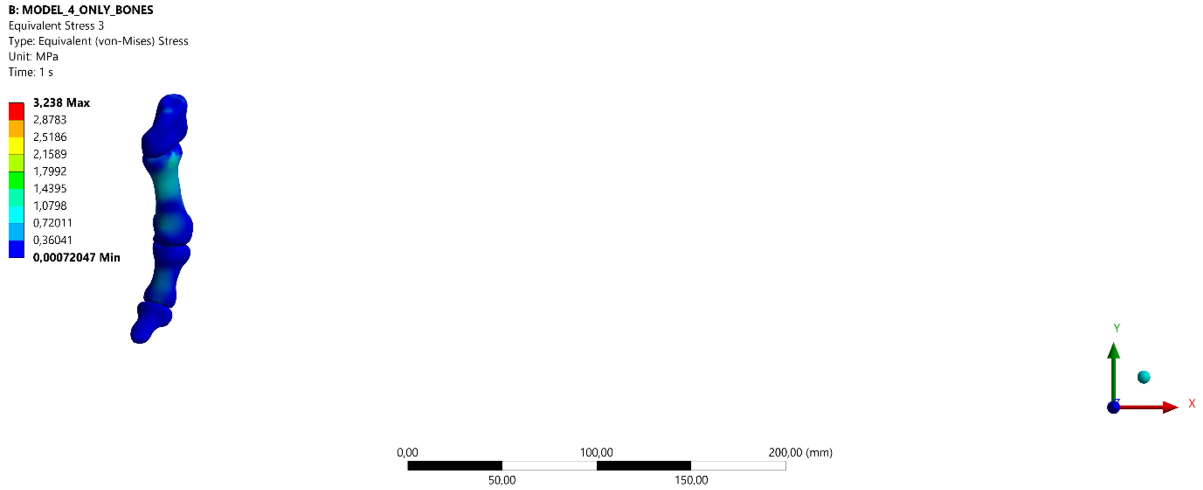

Type 4. Stress first bone.

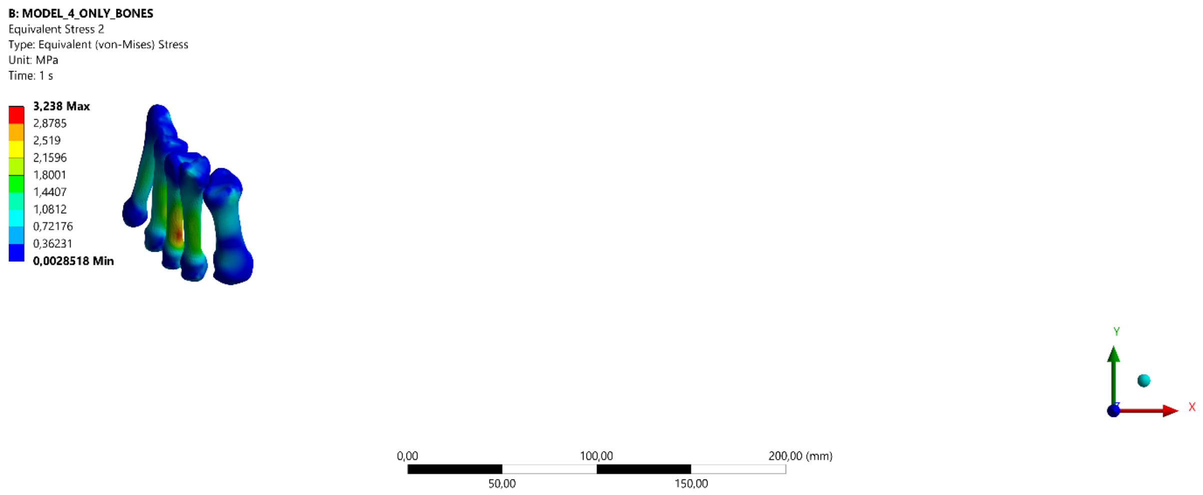

Type 4. Stress metatars.

Detailed finite element deformation distributions for the full foot model, first bone, and first metatarsal across all tibialis anterior tendon insertion types (Types 1–5).

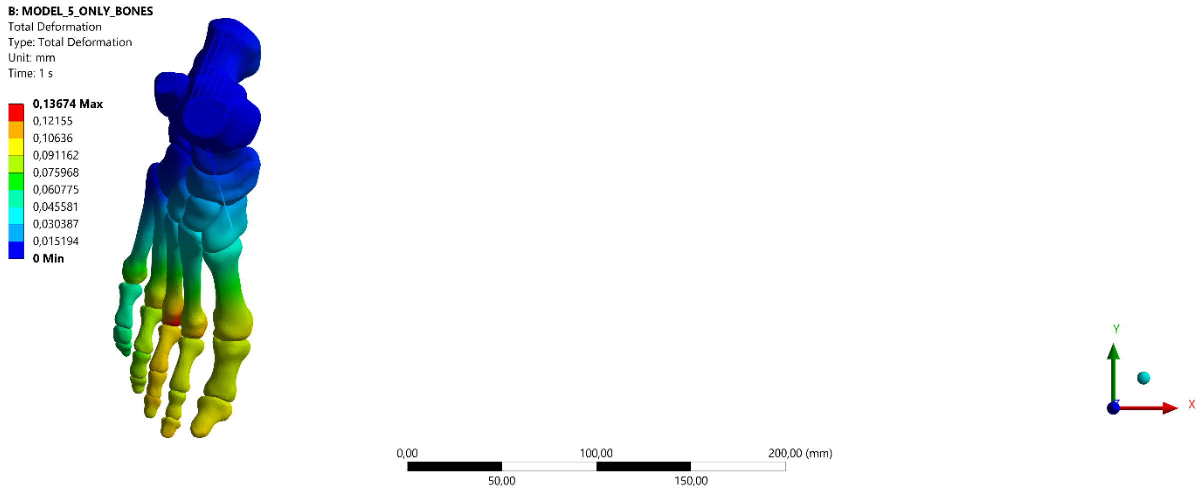

Type 5. Deformation.

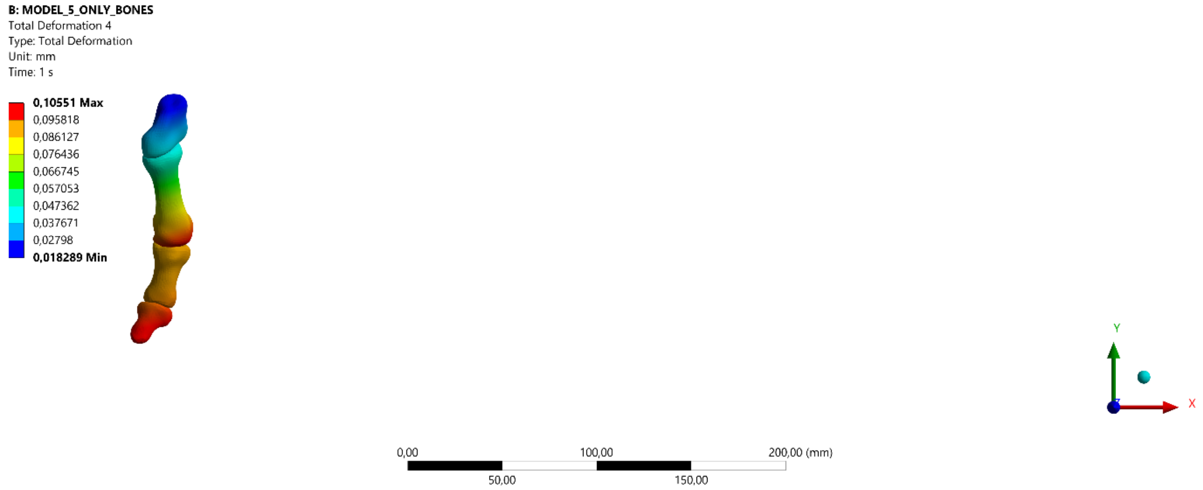

Type 5. Deformation first bone.

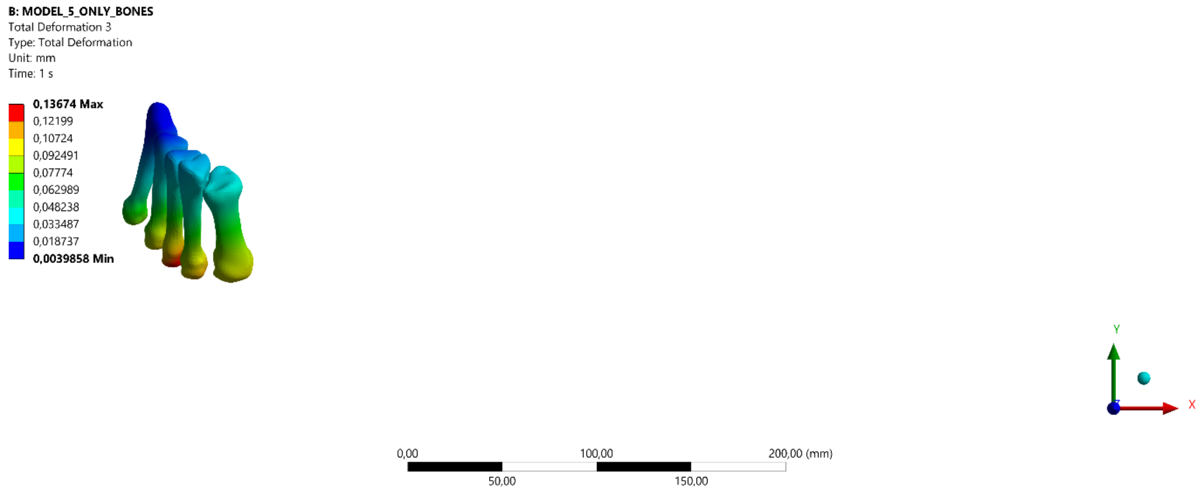

Type 5. Deformation metatars.

Detailed finite element deformation distributions for the full foot model, first bone, and first metatarsal across all tibialis anterior tendon insertion types (Types 1–5).

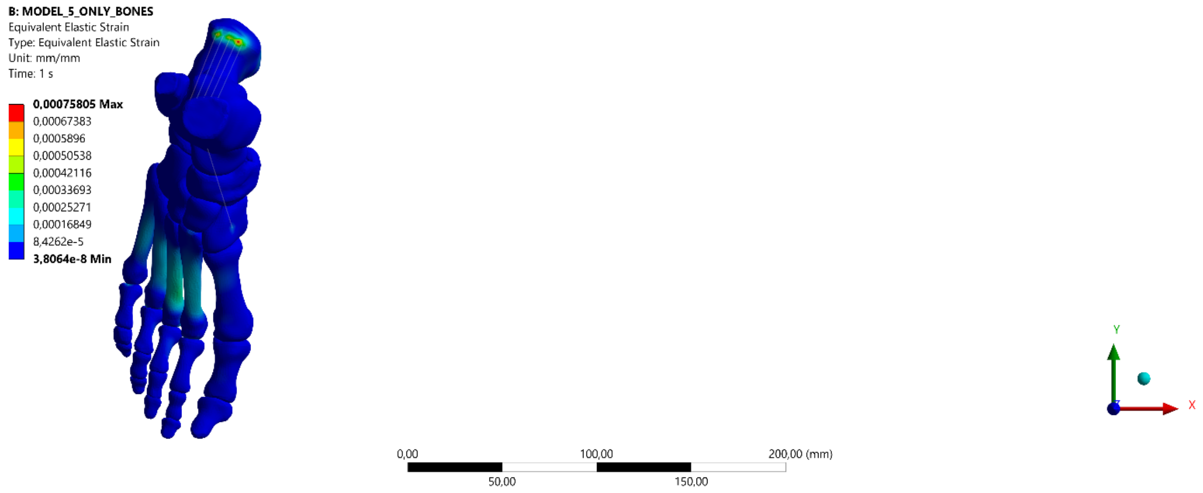

Type 5. Strain.

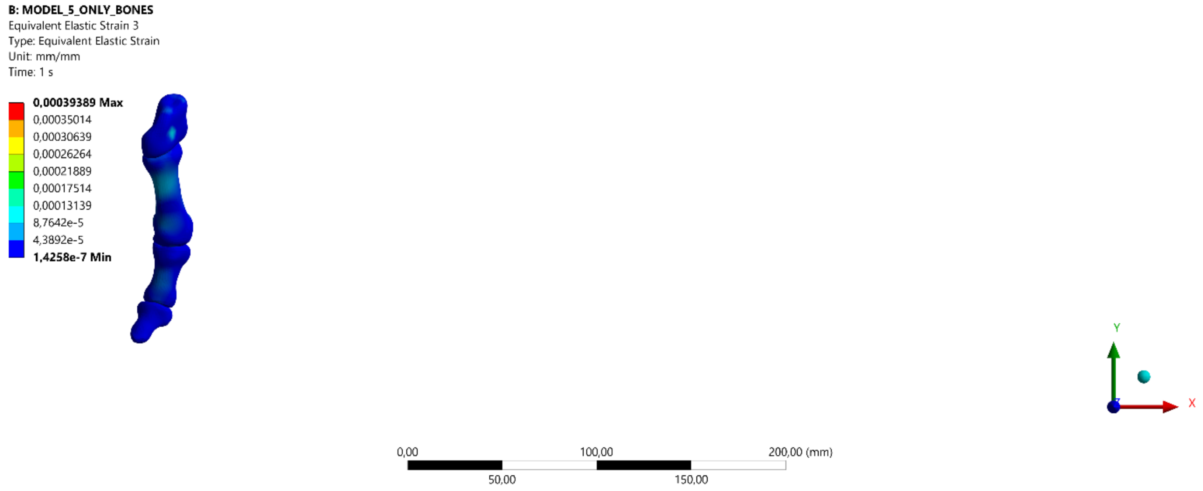

Type 5. Strain first bone.

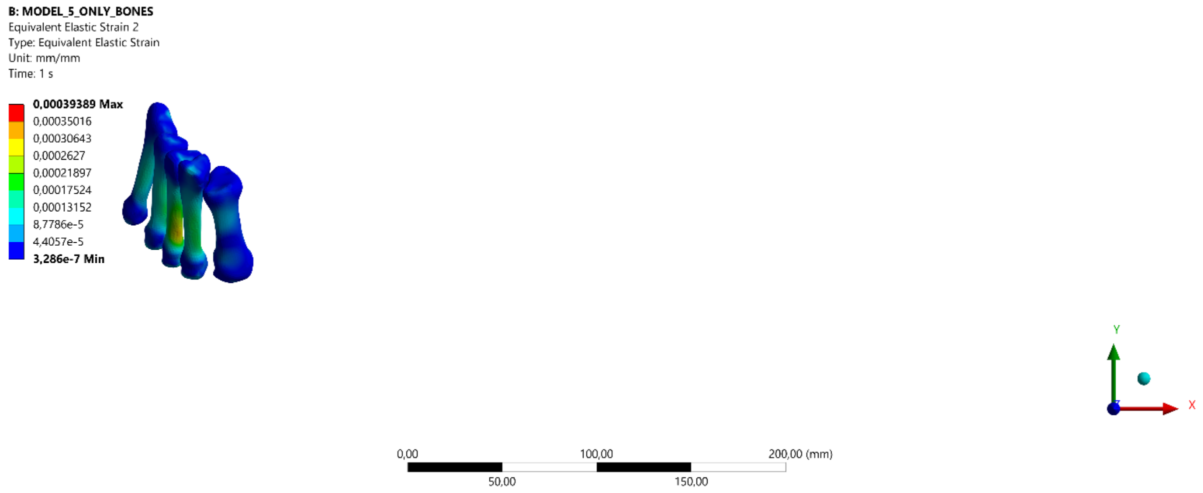

Type 5. Strain metatars.

Detailed finite element deformation distributions for the full foot model, first bone, and first metatarsal across all tibialis anterior tendon insertion types (Types 1–5).

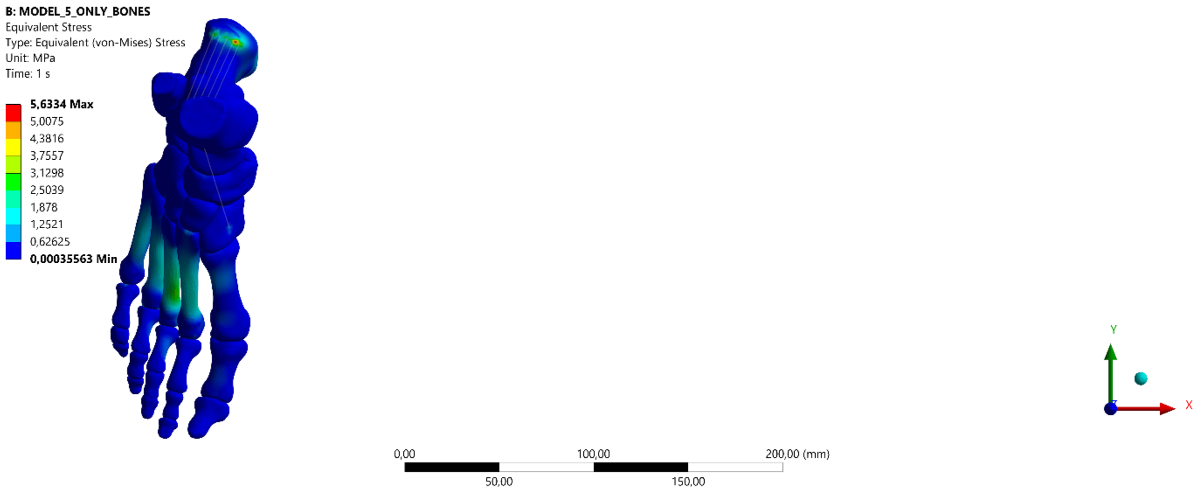

Type 5. Stress.

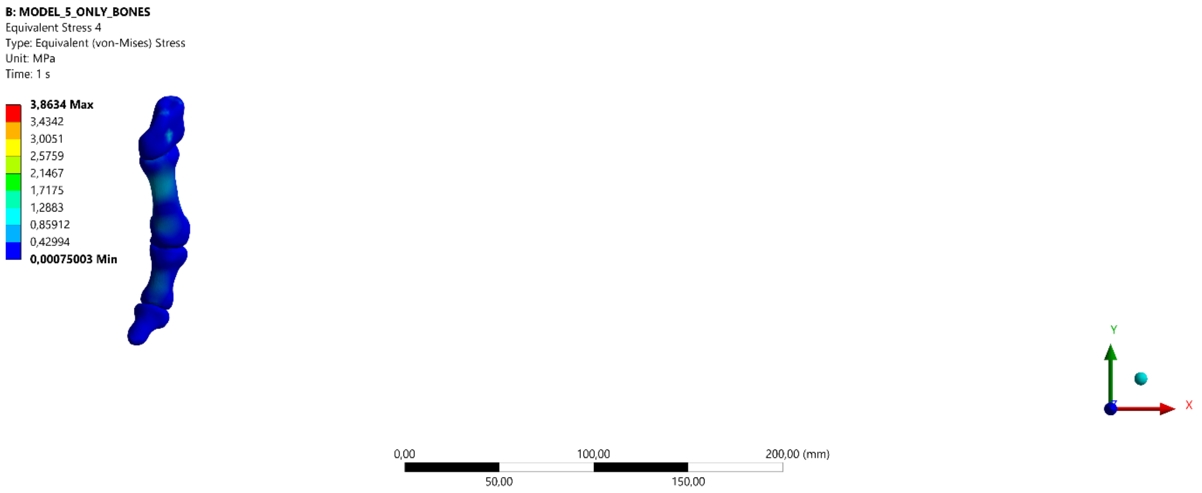

Type 5. Stress first bone.

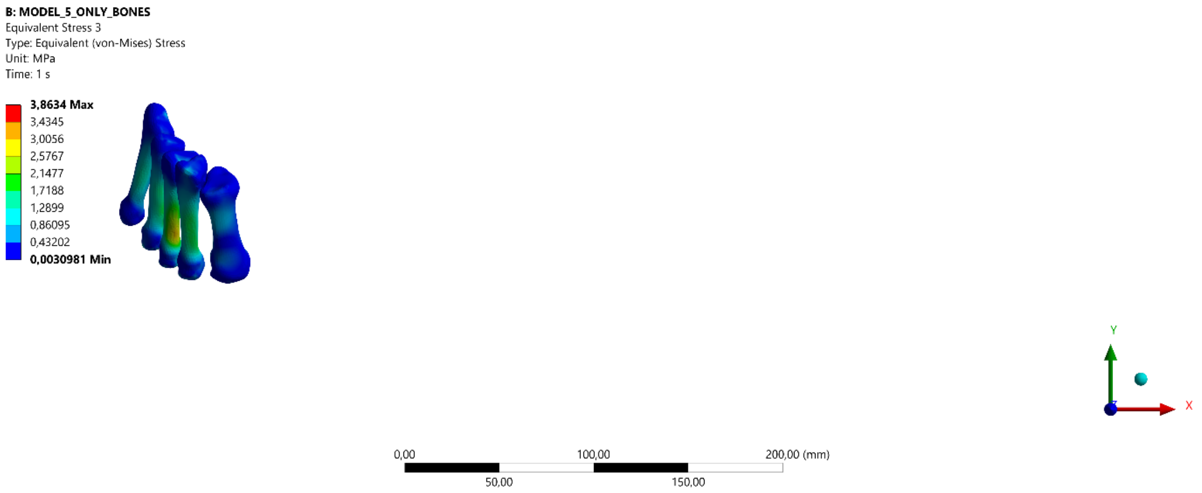

Type 5. Stress metatars.
